# Varimax Rotation Based on Gradient Projection Is a Feasible Alternative to SPSS

**DOI:** 10.3389/fpsyg.2019.00645

**Published:** 2019-03-26

**Authors:** Anneke Cleopatra Weide, André Beauducel

**Affiliations:** Department of Methods and Evaluation, Institute of Psychology, University of Bonn, Bonn, Germany

**Keywords:** gradient projection, Varimax, factor rotation, component rotation, principal component analysis, random start loadings, local optima

## Abstract

Gradient projection rotation (GPR) is an openly available and promising tool for factor and component rotation. We compare GPR toward the Varimax criterion in principal component analysis to the built-in Varimax procedure in SPSS. In a simulation study, we tested whether GPR-Varimax yielded multiple local solutions by creating population simple structure with a single optimum and with two optima, a global and a local one (double-optimum condition). The other conditions comprised the number of components (*k* = 3, 6, 9, and 12), the number of variables per component (*m/k* = 4, 6, and 8), the number of iterations per rotation (*i* = 25 and 250), and whether loadings were Kaiser normalized before rotation or not. GPR-Varimax was conducted with unrotated and multiple (*q* = 1, 10, 50, and 100) random start loadings. We found equal results for GPR-Varimax and SPSS-Varimax in most conditions. The few very small differences in favor of SPSS-Varimax were eliminated when Kaiser-normalized loadings and 250 iterations per rotation were used. Selecting the best solution out of multiple random starts in GPR-Varimax increased proximity to population components in the double-optimum condition with Kaiser normalized loadings, for which GPR-Varimax recovered population structure better than SPSS-Varimax. We also included an empirical example and found that GPR-Varimax and SPSS-Varimax yielded highly similar solutions for orthogonal simple structure in a real data set. We suggest that GPR-Varimax can be used as an alternative to Varimax rotation in SPSS. Users of GPR-Varimax should allow for at least 250 iterations, normalize loadings before rotation, and select the best solution from at least 10 random starts to ensure optimal results.

## Introduction

Exploratory factor analysis (EFA) and principal component analysis (PCA) are of major relevance in behavioral research, and many extraction and rotation methods have been proposed for them. Commercial statistical software, like SPSS or SAS, have implementations for the most popular extraction and rotation methods. Newer developments, like parallel analysis for extraction, Infomax, Geomin, and Partially Specified Target for rotation, can usually not be conducted by the built-in procedures in commercial software. However, some scientists have provided free software or code to be run in the non-commercial statistical software package R for these methods, making them accessible to a great audience. For example, Lorenzo-Seva and Ferrando (2006) have developed the software FACTOR with implementations for newer developments of factor analysis. Moreover, the Mplus software (Muthén and Muthén, 2015) also allows for several different methods of factor rotation as a basis for exploratory structural equation modeling. Most of the rotation methods that are meanwhile available attempt to find simple structure (Carroll, 1953; Kaiser, 1958; Hendrickson and White, 1964). Bernaards and Jennrich (2005) propose a generalized algorithm for analytic factor and component rotation that can approach most known rotation criteria toward simple structure by means of gradient projection (GPR). The approach is very promising because it unifies different rotation criteria under a single algorithm. Even more appealing is the fact that GPR can be performed in both commercial and non-commercial statistical software. GPR implementations of most rotation criteria are available online for SPSS Matrix, SAS PROC IML, Matlab, Splus, and R^1^. To this point, the mathematical background of the algorithm and its refinements have been comprehensively and extensively described (Jennrich, 2001, 2002; Bernaards and Jennrich, 2005). The authors also provide demonstrations on how to use GPR with exemplary loading matrices. However, when a new statistical procedure and the related code are publicly available and ready-to-use for data analysis, potential users should be able to lean on simulation studies to ensure their results depend on their data rather than the software algorithm applied. Therefore, we provide a simulation study on GPR toward the Varimax criterion (Kaiser, 1958) because it is one of the most popular and accepted rotation criteria toward orthogonal simple structure (Fabrigar et al., 1999; Browne, 2001) and it is implemented in most statistical software packages. The popularity of Varimax rotation can be demonstrated by hits in Google Scholar searches. Entering “Varimax” on Google Scholar on February 13, 2019 yielded approximately 203,000 hits, as compared to 6,050 for “Quartimax,” 3,780 for “Equamax,” and 184 entries for “Parsimax.” Even though perfect orthogonality is rather unlikely, Varimax also exceeds the popularity of oblique rotation criteria, such as Promax (43,100 hits), Oblimin (39,500 hits), or Quartimin (1,710 hits). As one may assume that the relative popularity of Varimax rotation is due to the lack of a backward limit for the time frame in this search, we performed an additional Google Scholar search for publications in the time frame between 2014 and 2019 (searched on 02/13/2019). For this time frame, we got 24,700 hits when entering “Varimax.” For “Geomin,” a more recent method for oblique and orthogonal rotation (Browne, 2001), we got 2,460 hits. Thus, Varimax rotation is still one of the most used rotation methods, even when many other alternative rotation methods are meanwhile available (Browne, 2001). However, scientists that use Varimax seem to rely on procedures that can be performed by pushing a button in commercial statistical software like SPSS or SAS. Entering the combination of “Varimax” and “SPSS” yielded 85,400 hits, and we got 21,800 hits for the combination of “Varimax” and “SAS” in Google Scholar (searched on 02/13/2019 for the complete time frame). Meanwhile, the combination of “Varimax” and “gradient projection” resulted in 128 hits (when “gradient projection” was entered with the quotation mark operator). Hence, GPR has not yet reached the popularity that could be expected given its promising simplicity and generalizability, and it faces the challenge of competing against readily available procedures in established software. In line with this, of the abovementioned 128 hits on Google Scholar for the combination of “gradient projection” and “Varimax,” 55 of them comprised a combination of “gradient projection,” “Varimax,” and “R project” (status on 02/13/2019). This indicates that GPR-Varimax is often used in R, whereas most scientists that use commercial software stick to pushing buttons to perform built-in, easily available procedures. Hence, testing GPR in simulation studies could promote its use in non-commercial software like R. Since SPSS is one of the most popular software tools for factor and component rotation and the Varimax criterion is one of the most popular rotation criteria, we compare the GPR algorithm for the Varimax criterion to the built-in SPSS procedure for Varimax rotation. As for the choice between factor analysis and PCA, we investigate GPR-Varimax performance for the rotation of components because the component model is simpler and does not require the estimation of error factors (Harman, 1967). Thereby, the present simulation does not depend on the precision of different methods for the estimation of factor loadings and communalities and focuses on the precision of the rotation method alone.

In order to understand the approach of the present simulation study, we give a brief description of the GPR algorithm in factor and component rotation (Jennrich, 2001, 2002; Bernaards and Jennrich, 2005). More detailed descriptions of the algorithm can be found elsewhere (Mulaik, 2010). The idea of GPR is that any rotation toward simple structure relies on an optimization (minimization or maximization) criterion, where constraints are placed on some parts of the optimization function. The gradient projection algorithm can be used to solve such constraint optimization problems. A rotation of an initial *m* ×*k* loading matrix *A* (e.g., unrotated loadings) is given by

(1) Λ=AT,

where *T* is a *k* ×*k* transformation matrix with columns of unit length (i.e., its column sums of squares add up to 1). Let Φ be the correlation matrix between rotated factors or components. For an orthogonal rotation, the rotated components are required to remain uncorrelated, such that the *k*(*k-1*) non-diagonal elements of Φ are constrained to be zero, resulting in

(2) Φ=T′T=I.

The optimization criterion *Q* in factor and component rotation is a function of the rotated loading matrix Λ and thereby a function of *T*, denoted by

(3) f(T)=Q(Λ).

For example, the Varimax criterion seeks to maximize the variance of squared loadings (Kaiser, 1958) and is therefore a function of the transformation matrix *T*. The algorithm searches for a minimum of *f*(*T*), such that the Varimax criterion would be the negative of *Q*(Λ). The algorithm uses the negative gradient G of *f*(T) = *Q*(Λ). For orthogonal rotation, it is given by

(4) $$G=A\prime \frac{\partial Q}{\partial \Lambda }.$$

Each rotation criterion has its own expression for ∂Q. The algorithm starts with an initial *T* from the manifold *T* that comply with the constraints. Then, *T* is moved by its negative gradient with step length *α* to find a matrix *M* by

(5) M=T−αG.

Next, *M* is projected back onto *T* by normalizing its columns to unit length. The projection is denoted by ∼*T.* After each step, the rotation criterion *f*(∼*T*) is evaluated and compared to the previous *f*(*T*). For a sufficiently small *α*, the algorithm is strictly descending, and

(6) f(~T)<f(T).

Then, *T* is replaced with ∼*T*, and the algorithm starts again. If *f* (~*T*) ≥ *f*(*T*), *α* needs to be reduced (e.g., halved), until *f* (~*T*) < *f*(*T*). The algorithm continues until it converges, that is, when *f*(*T*) becomes minimal.

For the initial loading matrix Λ = AT, a to-be-rotated loading matrix *A* and a start transformation matrix *T* need to be inserted. Usually, *A* are unrotated loadings from an initial factor or component extraction. For the start transformation matrix *T*, we can insert any matrix that complies with the constraints. For example, we can use the *k* ×*k* identity matrix (Jennrich, 2001) or a random matrix whose columns have unit length (Bernaards and Jennrich, 2005; Mulaik, 2010). For the latter, Bernaards and Jennrich (2005) recommend running the GPR algorithm several times with multiple random start matrices to identify local optima. The code for multiple random start matrices can be retrieved from http://www.stat.ucla.edu/research/gpa. Local optima are relevant in analytic rotation because the algorithms search a minimum on curvilinear, complex loss-functions with many ups and downs (Rozeboom, 1992). Hence, beginning with a particular start transformation matrix *T* does not guarantee to find the global optimum for the rotation criterion. Addressing this issue, multiple random starts are often used in demonstrations of analytic rotation algorithms. For example, Kiers (1994) used 20 random start matrices to present the Simplimax method, just like Browne (2001) in his comparative overview on different analytic rotation methods. Recently, Hattori et al. (2017) published a paper on Geomin rotation, in which they identified local optima by iterating across 100 random start matrices. If the GPR algorithm is to be established as a feasible alternative to other rotation algorithms, it needs to be shown that it does not stop at a local optimum but finds the global optimum of the rotation criterion instead. In the case of local optima, using multiple random start loading matrices Λ = AT should result in multiple solutions. Hence, we investigate whether using different start transformation matrices *T* (identity and multiple random matrices) in GPR-Varimax leads to the same or different results. If results are equal for different numbers of start loading matrices (resulting from different transformation matrices), it supports the notion that the GPR algorithm overcomes local optima and does not require a substantial amount of multiple starts for optimal results. If results differ between multiple start loading matrices, the global optimum should be found by trying multiple random start loadings and choosing the solution for which the Varimax criterion becomes maximal.

Kaiser (1958) proposes to perform a pairwise rotation of two factors or components toward the Varimax criterion. When all pairwise rotations of components are performed, the overall Varimax criterion is calculated. Then, a next cycle of rotations of all component pairs is performed, and it is checked whether the Varimax criterion has increased. This procedure is repeated until a convergence criterion is met or until a given number of cycles (i.e., a maximum number of iterations) is reached. This procedure is realized in conventional rotation software like SPSS, where the number of iterations can be specified. The number of iterations can also be specified for the GPR-Varimax algorithm, but there, the procedure is based on the gradient projection algorithm. However, the number of iterations of both rotation algorithms should not be confounded with the number of start loading matrices, which can only be specified in GPR-Varimax. Nevertheless, since the number of iterations may also affect the quality of results, we also investigate the effect of the number of iterations for GPR-Varimax and SPSS-Varimax.

Given the possibility of local optima, investigating GPR-Varimax in a simulation study requires population models that can adequately address the question of whether the algorithm finds the global optimum. Therefore, we will compare GPR-Varimax and SPSS-Varimax for two different population models. The first model is perfect orthogonal simple structure, which Varimax rotation is designed to recover (Kaiser, 1958). In the case of perfect orthogonal simple structure, there is a marked single (global) optimum for the Varimax criterion in the population. This single-optimum population model constitutes a relatively basic test for any Varimax algorithm, because local Varimax optima may only occur due to sampling error. However, if population components are weakly defined, that is, represented by few variables, the relative effect of sampling error on the loadings will be more pronounced. In this case, solutions become less stable across samples than for well-defined population components, which are represented by more variables (Guadagnoli and Velicer, 1988). Therefore, we will assess rotation performance in the single-optimum simple structure for three levels of component-definition with four, six, and eight variables per population component.

**FIGURE 1:**
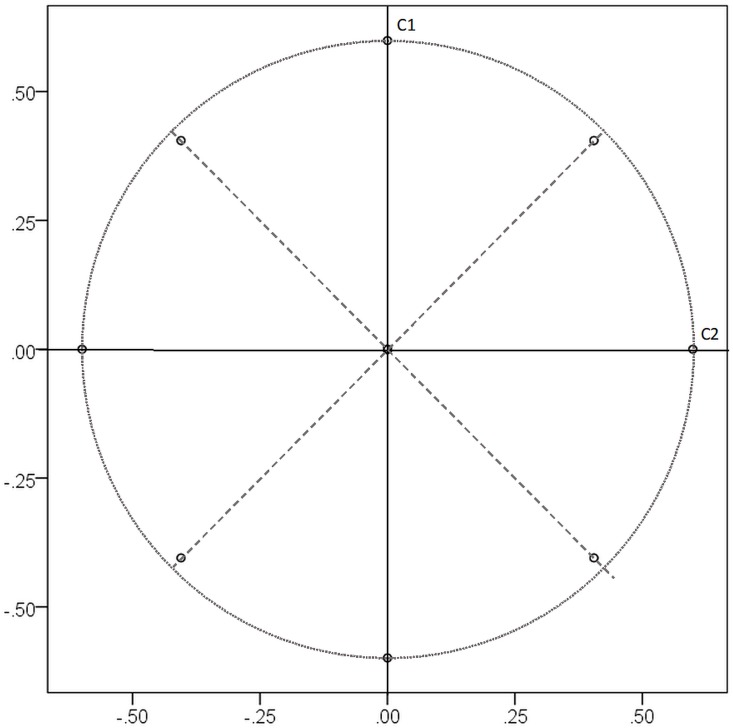
Loadings of 24 variables on component 1 (C1) and 2 (C2) of the global optimum solution (no Kaiser normalization) given in Table 2. The dotted circle marks the size of the largest main loadings on C1 and C2, the dashed lines mark the two components of the local optimum solution.

The second model is a double-optimum simple structure comprising a global and a local optimum. It is adapted from a circumplex model (Gurtman, 1992; Hofstee et al., 1992), where two or more competing simple structures exist in the population. These competing simple structures produce multiple local optima for the Varimax criterion. Figure 1 illustrates the loadings on two components of the global optimum solution as well as the two components of the local optimum solution in 45° angles. If one of the competing simple structures is recovered, one variable set has clear main loadings on only one component, whereas the other variable set has an ambiguous loading structure with main loadings on two components, and vice versa. For our purpose, the circumplex population model will be designed in a way that one of the competing simple structures has slightly higher loadings than the other one. For this solution, which represents the global optimum, the Varimax criterion becomes greater than for the competing solution, which represents a local optimum. Hence, there are two optima in the population, one of which is the global optimum and the other one is the local optimum for the Varimax criterion. Varimax rotation of sample data based on this population should on average recover the solution of the global optimum. However, if the GPR-algorithm sometimes stops at a local optimum for the Varimax criterion, these conditions could provoke the competing solution depending on the start loadings. Therefore, the double-optimum model constitutes a more challenging test of local optima in GPR-Varimax than the single-optimum model of perfect simple structure.

For both the single-optimum and the double-optimum population model, rotation performance of GPR-Varimax can be evaluated using the size of the Varimax criterion itself and by assessing proximity to the global optimum of the underlying population model. If GPR-Varimax produces a substantial amount of solutions with a local optimum, the Varimax criterion should differ between solutions depending on the start loadings inserted before rotation. However, using multiple random start loading matrices and selecting the solution for which the Varimax criterion becomes maximal could result in a larger Varimax criterion as compared to using only a single start loading matrix. If GPR-Varimax generally produces solutions of a global optimum, the Varimax criterion should not substantially differ depending on the start loadings. The same pattern should be found for proximity of the sample solutions to the global optimum of the underlying population model. Proximity to population models can be computed by congruence coefficients *c* of the components from the solution to the respective component in the population (Tucker, 1951; Korth and Tucker, 1975). Cut-off values have been suggested to assess similarity between factors or components by congruence coefficients. For example, Lorenzo-Seva and ten Berge (2006) state that values between *c* = 0.85 and *c* = 0.94 indicate fair similarity, and that components with a congruence of *c* ≥ 0.95 can be regarded as equal. Mundfrom et al. (2005) use different cut-offs with *c* ≥ 0.98 as excellent, values between *c* = 0.92 and *c* = 0.98 as good, values between *c* = 0.82 and *c* = 0.92 as borderline, values between *c* = 0.68 to *c* = 0.82 as poor, and congruence of *c* ≤ 0.68 indicating terrible agreement. We will evaluate congruence coefficients of the Varimax-rotated components (SPSS and GPR) with the population components using these suggestions.

To summarize, this simulation study investigates rotation performance of GPR-Varimax in comparison to SPSS-Varimax under a number of conditions (see below). In particular, we examine whether GPR-Varimax can overcome local optima by using a sufficient number of iterations and multiple start loadings for a single-optimum and a double-optimum model of simple structure in the population.

## Materials and Methods

We used IBM SPSS Version 25 and R Version 3.5.2 to double-check results for GPR-Varimax. GPR-Varimax was performed with the syntax code for SPSS matrix and the code of the R package *GPArotation*. The codes can be retrieved from http://www.stat.ucla.edu/research/gpa (Bernaards and Jennrich, 2005) or copied from the Supplementary Materials of this paper. We also performed the built-in Varimax-rotation of SPSS, which is available with the SPSS command *FACTOR* (SPSS-Varimax).

The conditions that we manipulated refer to the population model (single-optimum and double-optimum simple structure), the number of components (*k* = 3, 6, 9, and 12), the number of variables per component for the single-optimum model (*m/k* = 4, 6, and 8), sample size (*n* = 100 and *n* = 300), the number of iterations per rotation (*i* = 25 and 250), and whether Varimax-rotation was based on Kaiser normalized loadings or not. Moreover, four different numbers of random start loading matrices were investigated for GPR-Varimax (*q* = 1, 10, 50, and 100).

### Population Factor Models

We generated two population models based on common and unique factors. The first population model was a single-optimum model of perfect orthogonal simple structure. Main loadings for the single-optimum model were *a_0_* = 0.50 and zero on all other variables for population factors. Unique variances were $d_{0}=\sqrt{1-0.50^{2}}$ accordingly. We used *m/k* = 4, 6, and 8 variables per factor. Simple structure loadings of the population factors can be inspected in Table 1 for three factors and 24 variables (*m/k* = 6), together with the respective Varimax-rotated population components.

**Table 1:** Loading matrices of the single-optimum population model for three simple structure population factors and corresponding Varimax-rotated population components.

	Factors			Components					
				No Kaiser			With Kaiser		
	F1	F2	F3	C1	C2	C3	C1	C2	C3
V1	**0.50**	0.00	0.00	**0.58**	0.00	0.00	**0.58**	0.00	0.00
V2	**0.50**	0.00	0.00	**0.58**	0.00	0.00	**0.58**	0.00	0.00
V3	**0.50**	0.00	0.00	**0.58**	0.00	0.00	**0.58**	0.00	0.00
V4	**0.50**	0.00	0.00	**0.58**	0.00	0.00	**0.58**	0.00	0.00
V5	**0.50**	0.00	0.00	**0.58**	0.00	0.00	**0.58**	0.00	0.00
V6	**0.50**	0.00	0.00	**0.58**	0.00	0.00	**0.58**	0.00	0.00
V7	**0.50**	0.00	0.00	**0.58**	0.00	0.00	**0.58**	0.00	0.00
V8	**0.50**	0.00	0.00	**0.58**	0.00	0.00	**0.58**	0.00	0.00
V9	0.00	**0.50**	0.00	0.00	**0.58**	0.00	0.00	**0.58**	0.00
V10	0.00	**0.50**	0.00	0.00	**0.58**	0.00	0.00	**0.58**	0.00
V11	0.00	**0.50**	0.00	0.00	**0.58**	0.00	0.00	**0.58**	0.00
V12	0.00	**0.50**	0.00	0.00	**0.58**	0.00	0.00	**0.58**	0.00
V13	0.00	**0.50**	0.00	0.00	**0.58**	0.00	0.00	**0.58**	0.00
V14	0.00	**0.50**	0.00	0.00	**0.58**	0.00	0.00	**0.58**	0.00
V15	0.00	**0.50**	0.00	0.00	**0.58**	0.00	0.00	**0.58**	0.00
V16	0.00	**0.50**	0.00	0.00	**0.58**	0.00	0.00	**0.58**	0.00
V17	0.00	0.00	**0.50**	0.00	0.00	**0.58**	0.00	0.00	**0.58**
V18	0.00	0.00	**0.50**	0.00	0.00	**0.58**	0.00	0.00	**0.58**
V19	0.00	0.00	**0.50**	0.00	0.00	**0.58**	0.00	0.00	**0.58**
V20	0.00	0.00	**0.50**	0.00	0.00	**0.58**	0.00	0.00	**0.58**
V21	0.00	0.00	**0.50**	0.00	0.00	**0.58**	0.00	0.00	**0.58**
V22	0.00	0.00	**0.50**	0.00	0.00	**0.58**	0.00	0.00	**0.58**
V23	0.00	0.00	**0.50**	0.00	0.00	**0.58**	0.00	0.00	**0.58**
V24	0.00	0.00	**0.50**	0.00	0.00	**0.58**	0.00	0.00	**0.58**

*With Kaiser, Loadings were Kaiser normalized before rotation. No Kaiser, Non-normalized loadings were rotated. Main loadings are given in boldface. Population components were used to compute congruence coefficients with sample components obtained by GPR-Varimax and by SPSS-Varimax.*

The second population model was a double-optimum simple structure, where two competing simple structures (a stronger and a weaker one) existed in the population. We created the double-optimum structure for 2/3 of the variables and factors and kept perfect simple structure for the remaining 1/3 of the variables and factors for all conditions. For the 1/3 perfect simple structure variables and factors, main loadings remained at *a_0_* = 0.50 and unique variances at $d_{0}=\sqrt{1-0.50^{2}}$. We defined circumplexes of two factors, where the rotational position of two factors competed against another rotational position of the two factors. For this purpose, we used 16 variables per circumplex to balance out loadings. For each circumplex, main loadings were unique for 8 variables (*a_1_*/-*a_1_*), representing the stronger simple structure, and ambiguous for the other 8 variables (*a_2_*/-*a_2_*), representing the weaker competing simple structure. Ambiguous main loadings were created by distributing the main loadings onto two factors. The loadings for three factors with ambiguous simple structure are given in Table 2. For the conditions with *k* = 6, 9, and 12 factors, the population loading matrices were created by means of a blockwise repetition of the loading pattern presented in Table 2.

**Table 2:** Loading matrices of the double-optimum model for three simple structure population factors and corresponding Varimax-rotated population components.

	Factors			Components											
				No Kaiser						With Kaiser					
				Global optimum			Local optimum			Global optimum			Local optimum		
	F1	F2	F3	C1	C2	C3	C1	C2	C3	C1	C2	C3	C1	C2	C3
V1	**0.51**	0.00	0.00	**0.60**	0.00	0.00	**0.42**	**0.42**	0.00	**0.23**	**–0.55**	0.00	**0.55**	**–0.23**	0.00
V2	**0.51**	0.00	0.00	**0.60**	0.00	0.00	**0.42**	**0.42**	0.00	**0.23**	**–0.55**	0.00	**0.55**	**–0.23**	0.00
V3	**0.34**	**0.34**	0.00	**0.41**	**0.41**	0.00	**0.57**	0.00	0.00	**–0.22**	**–0.53**	0.00	**0.22**	**–0.53**	0.00
V4	**0.34**	**0.34**	0.00	**0.41**	**0.41**	0.00	**0.57**	0.00	0.00	**–0.22**	**–0.53**	0.00	**0.22**	**–0.53**	0.00
V5	0.00	**0.51**	0.00	0.00	**0.60**	0.00	**0.42**	**–0.42**	0.00	**–0.55**	**–0.23**	0.00	**–0.23**	**–0.55**	0.00
V6	0.00	**0.51**	0.00	0.00	**0.60**	0.00	**0.42**	**–0.42**	0.00	**–0.55**	**–0.23**	0.00	**–0.23**	**–0.55**	0.00
V7	**0.34**	**–0.34**	0.00	**0.41**	**–0.41**	0.00	0.00	**0.57**	0.00	**0.53**	**–0.22**	0.00	**0.53**	**0.22**	0.00
V8	**0.34**	**–0.34**	0.00	**0.41**	**–0.41**	0.00	0.00	**0.57**	0.00	**0.53**	**–0.22**	0.00	**0.53**	**0.22**	0.00
V9	0.00	**–0.51**	0.00	0.00	**–0.60**	0.00	**–0.42**	**0.42**	0.00	**0.55**	**0.23**	0.00	**0.23**	**0.55**	0.00
V10	0.00	**–0.51**	0.00	0.00	**–0.60**	0.00	**–0.42**	**0.42**	0.00	**0.55**	**0.23**	0.00	**0.23**	**0.55**	0.00
V11	**–0.34**	**–0.34**	0.00	**–0.41**	**–0.41**	0.00	**–0.57**	0.00	0.00	**0.22**	**0.53**	0.00	**–0.22**	**0.53**	0.00
V12	**–0.34**	**–0.34**	0.00	**–0.41**	**–0.41**	0.00	**–0.57**	0.00	0.00	**0.22**	**0.53**	0.00	**–0.22**	**0.53**	0.00
V13	**–0.51**	0.00	0.00	**–0.60**	0.00	0.00	**–0.42**	**–0.42**	0.00	**–0.23**	**0.55**	0.00	**–0.55**	**0.23**	0.00
V14	**–0.51**	0.00	0.00	**–0.60**	0.00	0.00	**–0.42**	**–0.42**	0.00	**–0.23**	**0.55**	0.00	**–0.55**	**0.23**	0.00
V15	**–0.34**	**0.34**	0.00	**–0.41**	**0.41**	0.00	0.00	**–0.57**	0.00	**–0.53**	**0.22**	0.00	**–0.53**	**–0.22**	0.00
V16	**–0.34**	**0.34**	0.00	**–0.41**	**0.41**	0.00	0.00	**–0.57**	0.00	**–0.53**	**0.22**	0.00	**–0.53**	**–0.22**	0.00
V17	0.00	0.00	**0.50**	0.00	0.00	**0.59**	0.00	0.00	**0.59**	0.00	0.00	**0.59**	0.00	0.00	**0.59**
V18	0.00	0.00	**0.50**	0.00	0.00	**0.59**	0.00	0.00	**0.59**	0.00	0.00	**0.59**	0.00	0.00	**0.59**
V19	0.00	0.00	**0.50**	0.00	0.00	**0.59**	0.00	0.00	**0.59**	0.00	0.00	**0.59**	0.00	0.00	**0.59**
V20	0.00	0.00	**0.50**	0.00	0.00	**0.59**	0.00	0.00	**0.59**	0.00	0.00	**0.59**	0.00	0.00	**0.59**
V21	0.00	0.00	**0.50**	0.00	0.00	**0.59**	0.00	0.00	**0.59**	0.00	0.00	**0.59**	0.00	0.00	**0.59**
V22	0.00	0.00	**0.50**	0.00	0.00	**0.59**	0.00	0.00	**0.59**	0.00	0.00	**0.59**	0.00	0.00	**0.59**
V23	0.00	0.00	**0.50**	0.00	0.00	**0.59**	0.00	0.00	**0.59**	0.00	0.00	**0.59**	0.00	0.00	**0.59**
V24	0.00	0.00	**0.50**	0.00	0.00	**0.59**	0.00	0.00	**0.59**	0.00	0.00	**0.59**	0.00	0.00	**0.59**

*With Kaiser, Loadings were Kaiser normalized before rotation. No Kaiser, Non-normalized loadings were rotated. Main loadings are given in boldface. Population components were used to compute congruence coefficients with sample components obtained by GPR-Varimax and by SPSS-Varimax.*

### Population Components and Data Sets

Since we investigated Varimax-rotation in PCA, we needed to obtain population components from the population factors of the models. Population components were then used to evaluate GPR-Varimax and SPSS-Varimax rotation of sample component loadings by means of congruence with the respective population components. To obtain population components, we generated population data sets on the basis of the population factor models and submitted these population data sets to PCA (see Tables 1, 2).

We followed a procedure described by Grice (2001) to generate finite population data sets based on common and unique factors. Thereby, we first used the SPSS Mersenne Twister random number generator to generate normally distributed, z-standardized random data *X* containing *k* + *m* preliminary factor scores. We then performed PCA on these data, again extracting *k* + *m* preliminary components without rotating them. Since component extraction in PCA is based on orthogonality, this step ensures that the final factors, common and unique, are all based on perfectly uncorrelated random variables to fulfill the requirements of population data. We saved the *k* + *m* component scores, which served as the *k* common factors *F* and *m* unique factor scores *U*. We then used an SPSS Matrix script in order to generate a finite population of observed variables by the common factor model Z = FA′ + UD′ (Gorsuch, 1983; Grice, 2001). The loading matrices *A* for common and *U* for unique factors were taken from the respective population factor models. The population data sets consisted of 100,000 cases for all populations that comprised 1,000 samples with *n* = 100 and 300,000 cases for all populations of 1,000 samples with *n* = 300. Finally, population component loadings were computed by performing a PCA with the built-in Varimax rotation in SPSS on the population data. Main loadings of population components for the single-optimum model were *a^∗^* = 0.58, for Varimax-rotation with and without Kaiser normalization (Table 1). Population component loadings for the double-optimum model differed between Varimax-rotation with and without Kaiser normalization (Table 2). Without Kaiser normalization, component loadings followed the modeled factor structure, where half of the double-optimum variables had unique main loadings, and the other half had ambiguous (double) main loadings. Thereby, we could identify the stronger simple structure (global optimum of the Varimax criterion) by submitting the population data set to PCA with SPSS-Varimax rotation of non-normalized loadings. For demonstration purposes, we also present the weaker simple structure (local optimum of the Varimax criterion, see Table 2). With Kaiser normalization, population component loadings from PCA with SPSS-Varimax did not follow the modeled structure with unique loadings for half of the double-optimum variables and ambiguous loadings for the other half. Instead, loadings were ambiguous for all double-optimum variables. This was the case for both the local and the global solution.

### Analysis of Simulated Sample Data

From the population data, 1,000 samples were drawn with either *n* = 100 or *n* = 300. Data for the samples were submitted to correlation-based PCA in SPSS for component extraction. We extracted a fixed number of components in the samples corresponding to the correct number of population components. Components of each sample were rotated with GPR-Varimax and SPSS-Varimax, both performed with and without Kaiser normalization (Kaiser, 1958). For initial loading matrices Λ = AT in GPR-Varimax, we used the *k* ×*k* identity matrix and random matrices as start transformation matrices *T*. For the computation of random start loading matrices for GPR-Varimax rotation, we used the SPSS Mersenne Twister random number generator in order to generate random transformation matrices. The unrotated component loading matrices *A* were post-multiplied by the random transformation matrices to generate random loading matrices. For the purpose of examining possible local solutions in GPR-Varimax, we allowed for different numbers of random start matrices with *q* = 1, 10, 50, and 100. Whenever more than a single random start matrix was used, we selected the solution with the maximum value for the Varimax criterion. This allowed us to investigate if GPR-Varimax yielded solutions with a local optimum depending on the start loadings, or if GPR-Varimax could find the global optimum independent of the start loading matrix. If rotation performance increased with a higher number of random start loading matrices that were used, this would indicate local optima. If rotation performance could not be improved by selecting the best solution out of multiple start loadings, and if the solution had the same quality as the corresponding SPSS-Varimax solution, this would indicate that the GPR-algorithm finds the global optimum.

We assessed rotation performance by computing the Varimax criterion (Kaiser, 1958) *v* for all rotations and by comparing GPR-Varimax loadings to population component loadings by means of congruence coefficients *c*. To compare and average results from multiple rotations, we had to solve the alignment problem in each solution, which refers to component reflection (the sign of the loadings) and component interchange (the position of each component in a given loading matrix Λ). We reflected loading signs by multiplying each column of Λ with the sign of the loading with the maximum absolute size of the respective column. We determined the position of each component in Λ by congruence coefficients *c* with population components. For each column in Λ, we selected the component that had the highest *c* with the respective population component of that position. These maximum congruence coefficients were then averaged across components in each sample, indicating proximity of the Varimax-rotated sample loadings to corresponding population component loadings (Mundfrom et al., 2005). In addition to proximity of the sample loadings to population loadings, we also computed deviation of the sample loadings from population component loadings for each solution. Therefore, we calculated the root mean square error (RMSE) based on the squared differences between rotated and population loadings (see Supplementary Materials). For each condition and method (GPR-Varimax, SPSS-Varimax, with and without Kaiser normalization), we averaged values for the three criteria (*v*, *c*, RMSE) of rotation performance across all 1,000 samples.

## Results

We compared rotation performance of GPR-Varimax and SPSS-Varimax on simulated data for the single-optimum and the double-optimum population model. We also included an analysis of real data to compare simple structure solutions from GPR-Varimax and SPSS-Varimax in an empirical setting.

### Cut-Offs for Equality of Results

For comparisons between GPR-Varimax and SPSS-Varimax, we used cut-offs defining equality of rotation performance. For this end, we decided on a conjunctive cut-off for the mean Varimax criterion and mean congruence with population loadings of solutions across 1,000 samples. Cut-offs were chosen to match the scaling of the values. The mean Varimax criterion in the samples ranged from *v* = 0.0066 (double-optimum, *k* = 12, *n* = 100, with Kaiser normalization) to *v* = 0.0417 (single-optimum, *k* = 3, *m/k* = 4, *n* = 300, with and without Kaiser normalization). Mean congruence coefficients in the samples ranged from *c* = 0.729 (single-optimum, *k* = 12, *m/k* = 4, *n* = 100, no Kaiser normalization) to *c* = 0.984 (single-optimum, *k* = 3, *m/k* = 8, *n* = 300, with and without Kaiser normalization). The exact values for the mean Varimax criterion *v* and the mean congruence coefficients with population loadings *c* can be inspected in the Supplementary Materials of this paper. Given the scaling of the values, we considered the quality of results to be equal when both the absolute difference between the mean Varimax criterion of the GPR-Varimax solutions and the mean Varimax criterion of the SPSS-Varimax solutions was | ∆ | < 0.0001 and the absolute difference between the respective mean congruence coefficients was | ∆ | < 0.001. In other words, the quality of GPR-Varimax and SPSS-Varimax solutions was considered equal when the Varimax criterion differed by less than 0.0001 and when population congruence differed by less than 0.001. Tables 3–6 show differences in mean Varimax criterion and mean population congruence between SPSS-Varimax and GPR-Varimax, on which evaluations of the equality or inequality of rotation performance were based. Differences in the tables are displayed as GPR-mean minus SPSS-mean, such that a positive sign indicates better performance of GPR-Varimax, a negative sign indicates better performance of SPSS-Varimax, and an em-dash (—) indicates equal performance. The tables show results for unrotated start loadings (start *T* = identity), a single random start loading matrix, and 10 random start loadings in GPR-Varimax, of which the solution with the maximum Varimax criterion was selected for each sample. Increasing the number of random starts in GPR-Varimax to 50 and 100 yielded no greater differences than those found for up to 10 random start loadings. Therefore, results for 50 and 100 random starts in GPR-Varimax are not displayed in the tables.

**Table 3:** Differences in Varimax criterion between GPR-Varimax and SPSS-Varimax component rotation for single-optimum simple structure.

			*m/k* = 4				*m/k* = 6				*m/k* = 8			
			25 iterations		250 iterations		25 iterations		250 iterations		25 iterations		250 iterations	
		GPR start	No	With	No	With	No	With	No	With	No	With	No	With
*k*	*N*	loadings	Kaiser	Kaiser	Kaiser	Kaiser	Kaiser	Kaiser	Kaiser	Kaiser	Kaiser	Kaiser	Kaiser	Kaiser
3	100	Unrotated	—	—	—	—	—	—	—	—	-0.002	-0.001	—	—
		Random 1	—	—	—	—	—	—	—	—	—	—	—	—
		Random 10	—	—	—	—	—	—	—	—	—	—	—	—
	300	Unrotated	—	—	—	—	—	—	—	—	—	—	—	—
		Random 1	—	—	—	—	—	—	—	—	—	—	—	—
		Random 10	—	—	—	—	—	—	—	—	—	—	—	—
6	100	Unrotated	—	—	—	—	—	—	—	—	—	—	—	—
		Random 1	—	—	—	—	—	—	—	—	—	—	—	—
		Random 10	—	—	—	—	—	—	—	—	—	—	—	—
	300	Unrotated	—	—	—	—	—	—	—	—	—	—	—	—
		Random 1	—	—	—	—	—	—	—	—	—	—	—	—
		Random 10	—	—	—	—	—	—	—	—	—	—	—	—
9	100	Unrotated	—	—	—	—	—	—	—	—	—	0.0001	—	0.0001
		Random 1	—	—	—	—	—	—	—	—	—	0.0001	—	0.0001
		Random 10	—	—	—	—	—	—	—	—	—	0.0001	—	0.0001
	300	Unrotated	—	—	—	—	—	—	—	—	—	—	—	—
		Random 1	—	—	—	—	—	—	—	—	—	—	—	—
		Random 10	—	—	—	—	—	—	—	—	—	—	—	—
12	100	Unrotated	—	—	—	—	—	—	—	—	—	—	—	—
		Random 1	—	—	—	—	—	—	—	—	—	—	—	—
		Random 10	—	—	—	—	—	—	—	—	—	—	—	—
	300	Unrotated	—	—	—	—	—	—	—	—	—	—	—	—
		Random 1	—	—	—	—	—	—	—	—	—	—	—	—
		Random 10	—	—	—	—	—	—	—	—	—	—	—	—

*GPR, gradient-projection based rotation. k, number of components. m/k, number of variables per component. With Kaiser, loadings were Kaiser normalized before rotation. No Kaiser, non-normalized loadings. Differences refer to the mean Varimax criterion of 1,000 samples and were computed as GPR-mean – SPSS-mean. Differences of |Δ|≥ 0.0001 are reported, otherwise results are considered equal (“—“). A positive sign means that GPR-Varimax was better than SPSS-Varimax and vice versa.*

### Simulation Results for Single-Optimum Simple Structure

For the single-optimum population model, rotation performance of GPR-Varimax and SPSS-Varimax was extremely similar, and we found only few very slight differences Δ between them (Tables 3, 4). Values for the mean Varimax criterion in the subset of data with single-optimum simple structure ranged from *v* = 0.0072 (*m/k* = 8, *k* = 12, *n* = 100) to *v* = 0.0417 (*m/k* = 4, *k* = 3, *n* = 300). The Varimax criterion was larger for samples of *n* = 300 than for samples of *n* = 100, and Kaiser normalization of loadings before rotation yielded slightly smaller values for the Varimax criterion in small samples (*n* = 100). Values for mean congruence coefficients ranged from *c* = 0.727 (*m/k* = 4, *k* = 12, *n* = 100, GPR-Varimax, no Kaiser, 25 iterations per rotation) to *c* = 0.984 (*m/k* = 8, *k* = 3, *n* = 300, with and without Kaiser). Congruence increased with sample size, where all mean congruences were larger than *c* = 0.900 in all conditions with *n* = 300. The tendencies in mean Varimax criterion and congruence were found for both SPSS-Varimax and GPR-Varimax rotation.

**Table 4:** Differences in congruence coefficients with population components between GPR-Varimax and SPSS-Varimax for single-optimum simple structure.

			*m/k* = 4				*m/k* = 6				*m/k* = 8			
			25 iterations		250 iterations		25 iterations		250 iterations		25 iterations		250 iterations	
		GPR start	No	With	No	With	No	With	No	With	No	With	No	With
*k*	*N*	loadings	Kaiser	Kaiser	Kaiser	Kaiser	Kaiser	Kaiser	Kaiser	Kaiser	Kaiser	Kaiser	Kaiser	Kaiser
3	100	Unrotated	—	—	—	—	—	—	—	—	-0.002	-0.001	—	—
		Random 1	—	—	—	—	—	—	—	—	—	—	—	—
		Random 10	—	—	—	—	—	—	—	—	—	—	—	—
	300	Unrotated	—	—	—	—	—	—	—	—	—	—	—	—
		Random 1	—	—	—	—	—	—	—	—	—	—	—	—
		Random 10	—	—	—	—	—	—	—	—	—	—	—	—
6	100	Unrotated	—	—	—	—	-0.001	—	—	—	—	—	—	—
		Random 1	—	—	—	—	—	—	—	—	—	—	—	—
		Random 10	—	—	—	—	—	—	—	—	—	—	—	—
	300	Unrotated	—	—	—	—	—	—	—	—	—	—	—	—
		Random 1	—	—	—	—	—	—	—	—	—	—	—	—
		Random 10	—	—	—	—	—	—	—	—	—	—	—	—
9	100	Unrotated	-0.001	—	-0.001	—	—	—	—	—	—	—	—	—
		Random 1	-0.001	—	-0.001	—	—	—	—	—	—	—	—	—
		Random 10	-0.001	—	-0.001	—	—	—	—	—	—	—	—	—
	300	Unrotated	—	—	—	—	—	—	—	—	—	—	—	—
		Random 1	—	—	—	—	—	—	—	—	—	—	—	—
		Random 10	—	—	—	—	—	—	—	—	—	—	—	—
12	100	Unrotated	-0.002	-0.002	—	—	-0.001	-0.001	—	—	-0.001	—	—	—
		Random 1	-0.002	-0.002	—	—	-0.001	-0.001	—	—	-0.001	—	—	—
		Random 10	-0.002	-0.002	—	—	-0.001	-0.001	—	—	—	—	—	—
	300	Unrotated	—	—	—	—	—	—	—	—	—	—	—	—
		Random 1	—	—	—	—	—	—	—	—	—	—	—	—
		Random 10	—	—	—	—	—	—	—	—	—	—	—	—

*GPR, gradient-projection based rotation. k, number of components. m/k = number of variables per component. With Kaiser, loadings were Kaiser normalized before rotation. No Kaiser, non-normalized loadings. Differences refer the mean congruence coefficients of 1,000 samples and were computed as GPR-mean – SPSS-mean. Differences of |Δ|≥ 0.001 are reported, otherwise results are considered equal (“—”). A positive sign means that GPR-Varimax was better than SPSS-Varimax and vice versa.*

Using the combined cut-offs for the Varimax criterion and congruence, we found equal results for SPSS-Varimax and GPR-Varimax in about 90% of the comparisons, i.e., in 259 out of 288 comparisons (86 out of 96 conditions) for the single-optimum model (see Tables 3, 4). Most differences were at the margin of the cut-offs (|Δ| = 0.0001| for *v* and |Δ| = 0.001| for *c*) and thus very small. Regarding the Varimax criterion, only 8 out of the 288 comparisons showed different results between GPR-Varimax and SPSS-Varimax, six of which were in favor of GPR-Varimax (Table 3). Moreover, using unrotated start loadings (i.e., an identity start transformation matrix) in GPR-Varimax resulted in equal values for the mean Varimax criterion for up to four decimals as testing several random start loadings and selecting the solution with the maximum Varimax criterion. In some cases, an unrotated start loading matrix yielded a greater Varimax criterion than the subsequent random start loadings, such that the solution for unrotated start loadings was selected. The few and small differences (16 Δ = -0.001, 7 Δ = -0.002; see Table 4) in mean congruence coefficients were all in favor of SPSS-Varimax. They were only found in small samples (*n* = 100). Moreover, they diminished when components were defined by more variables (*m/k* = 4 vs. 6 vs. 8) and when 250 iterations were allowed in each rotation (3 neg. Δ occurred) instead of 25 iterations (20 neg. Δ). Furthermore, when loadings were Kaiser normalized before rotation, there were fewer differences (7 neg. Δ) between GPR-Varimax and SPSS-Varimax as opposed to rotation of non-normalized loadings (16 neg. Δ). In respect of start loadings in GPR-Varimax, using random start loadings eliminated SPSS-GPR differences in congruence in four conditions. In the other six conditions with SPSS-GPR differences, they remained the same for up to 3 decimals independent of start loadings in GPR-Varimax (unrotated or random). This was also true when 50 and 100 random start loadings were tested to find the maximum value for the Varimax criterion.

### Simulation Results for Double-Optimum Simple Structure

For the double-optimum model with a local and a global optimum of the Varimax criterion in the population, we found more differences in rotation performance between GPR-Varimax and SPSS-Varimax than for the single-optimum population models (Tables 5, 6). The mean Varimax criterion in the subset of data with double-optimum simple structure ranged from *v* = 0.0066 (*k* = 12, *n* = 100, with Kaiser) to *v* = 0.0227 (*k* = 3, *n* = 100, no Kaiser). It decreased when loadings were Kaiser normalized before rotation. With regards to sample size, the Varimax criterion was smaller for *n* = 300 than for *n* = 100 when three or six components were rotated, but larger for *n* = 300 than for *n* = 100 for the rotation of nine and 12 components. Mean congruences were found between *c* = 0.725 (*k* = 12, *n* = 100, with Kaiser, SPSS-Varimax) and *c* = 0.936 (*k* = 3, *n* = 300, with Kaiser, GPR-Varimax with 10 random start loadings). They were overall larger in samples of *n* = 300 (with all *c* > 0.900) and when loadings were not Kaiser normalized before rotation.

**Table 5:** Differences in Varimax criterion between GPR-Varimax and SPSS-Varimax component rotation for double-optimum simple structure.

			25 iterations		250 iterations	
		GPR start	No	With	No	With
*k*	*N*	loadings	Kaiser	Kaiser	Kaiser	Kaiser
3	100	Unrotated	—	—	—	—
		Random 1	—	—	—	—
		Random 10	—	—	—	—
	300	Unrotated	—	—	—	—
		Random 1	—	—	—	—
		Random 10	—	0.0001	—	—
6	100	Unrotated	—	—	—	—
		Random 1	—	—	—	—
		Random 10	—	—	—	—
	300	Unrotated	—	—	—	—
		Random 1	—	—	—	—
		Random 10	—	0.0001	—	0.0001
9	100	Unrotated	—	—	—	—
		Random 1	—	—	—	—
		Random 10	—	—	—	—
	300	Unrotated	—	—	—	—
		Random 1	—	—	—	—
		Random 10	—	—	—	—
12	100	Unrotated	—	—	—	—
		Random 1	—	—	—	—
		Random 10	—	—	—	—
	300	Unrotated	—	—	—	—
		Random 1	—	—	—	—
		Random 10	—	—	—	—

*GPR, gradient-projection based rotation. k, number of components. With Kaiser, loadings were Kaiser normalized before rotation. No Kaiser, non-normalized loadings. Differences refer to the mean Varimax criterion of 1,000 samples and were computed as GPR-mean – SPSS-mean. Differences of |Δ|≥ 0.0001 are reported, otherwise results are considered equal (“—”). A positive sign means that GPR-Varimax was better than SPSS-Varimax and vice versa.*

**Table 6:** Differences in congruence coefficients with population components between GPR-Varimax and SPSS-Varimax for double-optimum simple structure.

			25 iterations		250 iterations	
		GPR start	No	With	No	With
*k*	*N*	loadings	Kaiser	Kaiser	Kaiser	Kaiser
3	100	Unrotated	—	0.001	—	0.001
		Random 1	—	0.017	—	0.017
		Random 10	—	0.017	—	0.017
	300	Unrotated	0.001	0.002	—	—
		Random 1	0.001	0.027	—	0.029
		Random 10	—	0.036	—	0.030
6	100	Unrotated	—	—	—	—
		Random 1	0.001	—	—	—
		Random 10	0.001	—	—	—
	300	Unrotated	—	—	—	—
		Random 1	—	0.016	—	0.030
		Random 10	—	0.023	—	0.032
9	100	Unrotated	-0.001	-0.001	—	—
		Random 1	-0.001	-0.001	—	—
		Random 10	-0.001	-0.001	—	—
	300	Unrotated	-0.001	—	-0.001	—
		Random 1	-0.001	0.027	-0.001	0.026
		Random 10	—	0.034	—	0.028
12	100	Unrotated	-0.001	-0.001	0.001	—
		Random 1	-0.001	-0.001	0.001	—
		Random 10	-0.001	-0.001	0.001	—
	300	Unrotated	—	—	—	—
		Random 1	—	0.026	—	0.027
		Random 10	—	0.031	—	0.029

*GPR, gradient-projection based rotation. k, number of components. With Kaiser, loadings were Kaiser normalized before rotation. No Kaiser, non-normalized loadings. Differences refer to the mean congruence coefficients with population components of 1,000 samples and were computed as GPR-mean – SPSS-mean. Differences of |Δ|≥ 0.001 are reported, otherwise results are considered equal (“—”). A positive sign means that GPR-Varimax was better than SPSS-Varimax and vice versa.*

Results varied more between rotation methods (GPR-Varimax vs. SPSS-Varimax) and between start loadings in GPR-Varimax than in the single-optimum case. Out of 96 comparisons, rotation performance was equal in 50 comparisons, better for GPR-Varimax in 30 comparisons, and better for SPSS-Varimax in 16 comparisons (Tables 5, 6). Considering the mean Varimax criterion, GPR-Varimax with at least 10 random start loadings performed slightly better than SPSS-Varimax and GPR-Varimax with a single start loading matrix in three conditions (Table 5). However, greater variation was found when mean congruence coefficients were compared for up to three decimals (Table 6). Differences in favor of SPSS-Varimax were very small (Δ = -0.001). All negative differences were found for the rotation of nine and 12 components, and most of them occurred when 25 iterations per rotation were allowed, sample size was small, and loadings were not Kaiser normalized before rotation. These negative differences diminished when we increased the number of iterations per rotation from 25 (14 neg. Δ) to 250 (2 neg. Δ)or when sample size increased from *n* = 100 (16 neg. Δ) to *n* = 300 (4 neg. Δ). As in the single-optimum models, combining Kaiser normalized loadings and 250 iterations per rotation yielded no differences in favor of SPSS. Most differences in favor of GPR-Varimax were greater than those in favor of SPSS-Varimax and up to Δ = 0.036. All relevant differences of Δ ≥ 0.020 occurred in large samples with Kaiser normalized loadings. In these conditions, it was advantageous to use random start loadings rather than unrotated start loadings in GPR-Varimax. Highest congruence was reached when 10 or more random start loadings were used. Thereby, the mean Varimax criterion increased as well, but to a lesser extent than congruence with population components.

### Empirical Example Based on a Short Knowledge Test

In addition to the simulation study, we also analyzed real data from an empirical study to show the similarity of GPR-Varimax and SPSS-Varimax in an investigative application. Therefore, PCA was performed for 17 newly developed items of a knowledge test. A total sample of 397 voluntary participants (55 females; age in years, *M* = 19.53, *SD* = 2.49) from a German school worked for about 30 min on the 17 single choice knowledge items. Each item had one correct solution and four distractors. Six items were from the knowledge domain Geography/History, five items were from the knowledge domain Science, and six items were from the knowledge domain of Culture and Arts. The item inter-correlation matrix is given in the Supplementary Material (Supplementary Table S14). Three components from the unrotated PCA explaining 30.52% of the total variance were retained for Varimax rotation (for eigenvalues of the unrotated PCA, see Table 7). Varimax rotation was performed by means of SPSS-Varimax and GPR-Varimax with a maximum of 250 iterations. As a start transformation matrix in GPR-Varimax, we used the identity matrix, one random start matrix, and 10 random start matrices. We did not use more than 10 random start matrices because the results from the simulation study yielded that using more than 10 random start matrices for the rotation of three components did not improve rotation performance.

**Table 7:** Three Varimax-rotated principal components from a short knowledge test.

	GPR no Kaiser			GPR with Kaiser			SPSS with Kaiser		
	G/h	S	C	G/h	S	C	G/h	S	C
Geo/his 1	**0.51**	0.09	-0.12	**0.52**	0.11	-0.09	**0.52**	0.11	-0.09
Geo/his 2	**0.45**	-0.04	0.06	**0.45**	-0.02	0.09	**0.45**	-0.02	0.09
Geo/his 3	**0.59**	-0.03	0.25	**0.58**	-0.02	0.28	**0.58**	-0.02	0.28
Geo/his 4	**0.59**	0.01	-0.16	**0.59**	0.03	-0.13	**0.59**	0.03	-0.13
Geo/his 5	**0.46**	0.08	0.17	**0.45**	0.09	0.19	**0.45**	0.09	0.19
Geo/his 6	0.24	0.06	**0.48**	0.21	0.06	**0.49**	0.21	0.06	**0.49**
Science 1	0.11	**0.46**	0.07	0.09	**0.46**	0.09	0.09	**0.46**	0.09
Science 2	0.22	**0.38**	0.25	0.20	**0.38**	0.27	0.20	**0.38**	0.27
Science 3	0.20	**0.62**	0.15	0.17	**0.63**	0.17	0.17	**0.63**	0.17
Science 4	-0.10	**0.77**	0.02	-0.12	**0.76**	0.03	-0.12	**0.76**	0.03
Science 5	-0.01	**0.64**	-0.06	-0.02	**0.64**	-0.04	-0.02	**0.64**	-0.04
Culture 1	0.18	0.02	**0.32**	0.16	0.02	**0.33**	0.16	0.02	**0.33**
Culture 2	-0.09	0.04	**0.63**	-0.13	0.02	**0.63**	-0.13	0.02	**0.63**
Culture 3	-0.06	0.21	**0.31**	-0.09	0.20	**0.31**	-0.09	0.20	**0.31**
Culture 4	0.11	00.01	**0.51**	0.08	0.01	**0.51**	0.08	0.01	**0.51**
Culture 5	0.23	0.07	**0.43**	0.20	0.07	**0.45**	0.20	0.07	**0.45**
Culture 6	-0.14	0.17	**0.42**	-0.16	0.16	**0.42**	-0.16	0.16	**0.42**

*SPSS, rotation by means of the SPSS command FACTOR. GPR, gradient-projection based rotation. The GPR-Varimax solutions are displayed for unrotated loadings as start loadings. Loadings with an absolute size greater or equal 0.30 are given in boldface. First nine eigenvalues of unrotated PCA: 2.43, 1.52, 1.24, 1.17, 1.11, 1.00, 0.97, 0.96, 0.87.*

Although several secondary loadings occurred, the component loadings could be clearly interpreted in terms of the three knowledge domains. The congruence of the Varimax loadings based on SPSS-Varimax and Kaiser normalization (see Table 7) with the Varimax loadings from SPSS-Varimax without Kaiser normalization was 0.998. Thus, these two SPSS-Varimax loading matrices were nearly identical, so that it was sufficient to compare GPR-Varimax loadings only with the SPSS-Varimax loadings based on Kaiser normalization. Table 7 displays GPR-Varimax loadings where the start loading matrix were unrotated loadings (i.e., the transformation matrix for transforming the unrotated loading matrix into a start loading matrix was identity). It turned out that the loadings of GPR-Varimax with and without Kaiser normalization were nearly identical to the loadings resulting from Varimax rotation based on the SPSS FACTOR command and Kaiser normalization (see Table 7). The congruence of GPR-Varimax loadings based on Kaiser normalization with the corresponding SPSS-Varimax loadings based on Kaiser normalization were all greater than 0.999. The congruence of GPR-Varimax loadings without Kaiser normalization with the corresponding SPSS-Varimax loadings based on Kaiser normalization were all greater than 0.990. Thus, the different methods of Varimax rotation did not result in relevant differences of the loadings. These congruence coefficients were similar when one or 10 random start loading matrices were used for GPR Varimax. Overall, the Varimax criterion of all solutions (GPR-Varimax with and without Kaiser normalization, with unrotated start loadings, one and 10 random start matrices, and with SPSS without Kaiser normalization) was about 0.19 with small differences at the third decimal place. Given these results, it follows clearly that the interpretation of the component loadings was not altered by the different methods of Varimax rotation.

## Discussion

We assessed rotation performance of Varimax rotation in PCA based on gradient projection in comparison to the built-in SPSS-Varimax rotation in a simulation study. Referring to the discussion of multiple local optima in analytic factor and component rotation (Rozeboom, 1992; Bernaards and Jennrich, 2005; Mulaik, 2010), we examined whether the GPR-Varimax algorithm could find the global optimum of the Varimax criterion for a single-optimum and a double-optimum simple structure in the population. Therefore, we evaluated rotation performance of GPR-Varimax using unrotated loadings and multiple random start loadings. Moreover, we investigated whether selecting the best out of multiple random starts improved rotation performance of GPR-Varimax. Furthermore, we included an empirical example in which we compared Varimax solutions of GPR and SPSS for the analysis of real data.

### Summary and Interpretation of Results

We found that GPR-Varimax rotation was comparable to SPSS-Varimax rotation in both simulated and real data. Comparing rotation performance for the single-optimum and double-optimum population model in the simulation study yielded equal results in most manipulated conditions. When there was a single marked optimum of orthogonal simple structure in the population, results were extremely similar for GPR-Varimax and SPSS-Varimax. The combined comparison of the mean Varimax criterion for up to four decimals and mean congruence coefficients with population loadings for up to three decimals yielded equal results for 86 out of 96 conditions or 259 out of 288 comparisons. When sample size was *n* = 300, all comparisons showed equal results for GPR-Varimax and SPSS-Varimax. In smaller samples (*n* = 100), we found a few very small differences, most of which were comprised by slightly higher congruence coefficients of SPSS-Varimax solutions with population loadings (Δ = -0.001). Most of these differences occurred when components were weakly defined (*m/k* = 4 vs. *m/k* = 6 vs. *m/k* = 8). However, rotating Kaiser normalized loadings and allowing for 250 iterations per rotation eliminated all differences in favor of SPSS in small samples as well. Thus, with the right settings, the GPR-Varimax algorithm reaches an optimum for the Varimax criterion that yields results equal to SPSS-Varimax even in small samples and for weakly defined components. Furthermore, all differences in favor of SPSS-Varimax were extremely small and probably not relevant in applied contexts. Hence, even with 25 iterations per rotation and non-normalized loadings, GPR-Varimax can be expected to recover single-optimum population simple structure to a similar degree as SPSS for the presented conditions. Regarding multiple local solutions, we found little evidence that using multiple random start loadings in GPR-Varimax resulted in substantially different solutions for the single-optimum population model. Only in four conditions, it was slightly advantageous to use random start loadings rather than the unrotated loading matrix. In the other 92 conditions, using unrotated start loadings in GPR-Varimax was sufficient to match SPSS-Varimax performance.

When there were two optima, a global and a local one, of population simple structure, we found more differences between GPR-Varimax and SPSS-Varimax. However, more than half of the comparisons (50 out of 96) yielded equal results. Again, the Varimax criterion was the same in almost all comparisons (93 out of 96) for up to four decimals, but we found more differences in congruence coefficients with population components for up to three decimals. All differences in favor of SPSS (16 neg. Δ) were very small (Δ = -0.001), whereas those in favor of GPR (30 pos. Δ) were more substantial (50% of pos. Δ were larger than 0.020). As in the single-optimum case, all differences in favor of SPSS-Varimax were eliminated when Kaiser normalized loadings were rotated and 250 iterations were allowed in each rotation. All substantial differences in favor of GPR-Varimax occurred in samples of *n* = 300 and when loadings were Kaiser normalized before rotation. Thereby, GPR-Varimax solutions showed highest similarity to population components when at least 10 random start loading matrices were used. However, using more than 10 random start loadings did not increase congruence coefficients any further. Nevertheless, the results showed that GPR-Varimax solutions with a similar Varimax criterion as the SPSS-Varimax solution (|Δ| < 0.0001) produced by using 10 or more random start loading matrices with Kaiser normalized loadings led to relevant increases in congruence with population components. Thus, when there is a local optimum in the population loadings, GPR-Varimax may result in solutions that are more similar to the global optimum than SPSS-Varimax, while the Varimax criterion is similar or equal to SPSS-Varimax. Hence, when population components have substantial ambiguity, using multiple random start loadings in GPR-Varimax could increase the chance of approximating the population components with the global optimum. This unexpected finding could possibly be explained by the different approaches of maximizing the Varimax criterion by the built-in SPSS procedure as opposed to the GPR algorithm. The original Varimax procedure that is realized in SPSS analytically maximizes the Varimax criterion using derivatives for pairs of factors or components (Kaiser, 1958). In each iteration, the Varimax criterion of all component pairs is maximized. Although there are also iterations in order to maximize the Varimax criterion in GPR-Varimax, this algorithm is not based on pairwise rotations of components. Additional maximizing is performed when GPR-Varimax is conducted with multiple start loading matrices. In GPR-Varimax based on random start loading matrices, the complete loading matrices based on two different start loading matrices are compared and the loading matrix with the larger Varimax criterion is retained for the next step. Thus, a main difference between the SPSS-Varimax algorithm and the GPR-Varimax algorithm is that the latter does not rely on pairwise rotation of components in order to maximize the Varimax criterion. This should be investigated in further studies. However, the present results may indicate that a maximization of the Varimax criterion for the complete loading matrix might be less susceptible to sampling error variance than an algorithm that is based on component pairs.

Considering the results from both the single-optimum and the double-optimum population models, GPR-Varimax and SPSS-Varimax performed very similar in recovering population components. Therefore, mean congruence coefficients with population can be evaluated for the manipulated conditions for both rotation methods combined. According to the thresholds suggested by Lorenzo-Seva and ten Berge (2006), most solutions discovered by GPR-Varimax and SPSS-Varimax displayed fair similarity to population components with *c* > 0.85 (see Supplementary Materials). This was true for all conditions with *n* = 300. In the single-optimum case, all conditions with *n* = 300 except for *k* = 12 with *m/k* = 4 even yielded population congruence of *c* > 0.92, which has been suggested as threshold for *good* agreement by Mundfrom et al. (2005). For the other conditions, GPR-Varimax and SPSS-Varimax solutions fell into the range of *borderline* and *poor* agreement with population components, mostly depending on the number of components to be rotated, Kaiser normalization, and the population model beyond the simulated sample data sets. None of the mean congruence coefficients with population components were smaller than *c* = 0.68, as the threshold for *terrible* agreement (Mundfrom et al., 2005).

Conducting Varimax rotation by GPR and SPSS in PCA on a real data set supported the results of the simulation study. For the 17 newly developed items of a short knowledge test, both GPR-Varimax and SPSS-Varimax indicated a clear three-component solution. The recovered loading pattern was highly similar with congruence of *c* ≥ 0.990 between SPSS- and GPR-components, indicating *excellent agreement* between loading patterns (Mundfrom et al., 2005) or rather *equal* components (Lorenzo-Seva and ten Berge, 2006). Furthermore, the solutions did not differ between multiple start loadings in GPR-Varimax. Thus, it was not necessary to transform the unrotated start loadings from the data into random start loadings before Varimax-rotation by GPR. These findings support the notion that GPR-Varimax can be used as an alternative to SPSS-Varimax in empirical applications.

### Choices of Simulated Conditions

The results of the simulation study indicated that rotation performance in terms of similarity to population components foremost depended on the manipulated conditions rather than the rotation algorithm applied (SPSS-Varimax vs. GPR-Varimax). As the range from *poor* to *excellent* agreement (Mundfrom et al., 2005) with population components was covered by the presented conditions and hardly differed between GPR-Varimax and SPSS-Varimax, we consider the search space to be sufficiently exhausted. We used two population models to address the question of local optima. In the single-optimum model, we manipulated the *m/k* ratio (*m/k* = 4, 6, and 8) to test whether solutions remained stable when components were weakly defined (Guadagnoli and Velicer, 1988). The double-optimum model with a global and a local optimum of simple structure in the population was prone to produce local optima for the rotation of sample data if the GPR algorithm would stop at a local optimum instead of the global one. Regarding sample size, we used *n* = 100 as the minimum sample size recommended for factor analysis in the literature (Gorsuch, 1983). The second sample size of *n* = 300 exceeds most recommendations for minimum sample sizes and has been classified as *good* (MacCallum et al., 1999). Conducting PCA in even smaller samples would not be advisable, especially for a large number of components (here: maximum of 12) and variables (here: maximum of 96). The number of components for which we investigated performance of GPR-Varimax covers the numbers of facets embedded in the most popular theories on personality. The main discussion revolves around whether personality consists of three (Eysenck, 1991; Eysenck et al., 1992), five (Costa and McCrae, 2006), or six (Lee and Ashton, 2004) independent components or factors. Moreover, investigating rotation performance for up to 12 components, exceeds the number of components or factors investigated in most simulation studies on factor and component analysis (Guadagnoli and Velicer, 1988; MacCallum et al., 1999; Mundfrom et al., 2005; Trendafilov and Jolliffe, 2006). Furthermore, results for the manipulated conditions did not differ to a substantial degree (|Δ| < 0.001 for congruence and |Δ| < 0.0001 for the Varimax criterion) when more than 10 random start loading matrices in GPR-Varimax were used. Therefore, we decided to conduct all analyses for a maximum of 100 random start loadings in GPR-Varimax and report results for up to 10. Using a maximum of 100 random start loadings is also in line with findings by Hattori et al. (2017), who found that using 100 multiple starts sufficed to examine local solutions in Geomin rotation. It also exceeds the number of random starts performed in other demonstrations of factor rotation where multiple local optima were of concern (Kiers, 1994; Browne, 2001; Trendafilov and Jolliffe, 2006).

### Future Research

Future research should investigate the advantage of random starts in GPR-Varimax we found for Kaiser normalized loadings in the double-optimum case in more detail. In particular, it would be interesting to isolate the effect of random start loadings from the effect of the GPR algorithm. Therefore, one could introduce random start loadings in SPSS by writing a syntax for the SPSS MATRIX environment. These random start loadings should then be inserted in the built-in SPSS-Varimax procedure that is available by the command FACTOR to produce multiple solutions. In the same way as we proceeded with GPR-Varimax, the solutions should be successively replaced to select the one with the maximum Varimax criterion. If congruence with population components thereby reaches the level that we found for GPR-Varimax, the effect can be attributed to random starts rather than the Varimax algorithm applied. If differences between GPR-Varimax and SPSS-Varimax remain, the effect can be attributed to either the GPR-Varimax algorithm alone or a combination of multiple random starts and differences in the Varimax procedure between GPR and SPSS.

Moreover, it would be interesting to extend the investigation from rotation in PCA to rotation in EFA to examine whether GPR-Varimax performs similarly when different extraction methods are applied. For example, GPR-Varimax could be applied to loadings obtained from maximum likelihood factor analysis or principal axis factoring to investigate whether rotation performance remains stable when unique variances need to be estimated as well (Harman, 1967). Furthermore, one could introduce more complex loading structures in the single- and double-optimum population models. For example, one could manipulate component/factor saturation (Guadagnoli and Velicer, 1988) by varying the size of the main loadings (here *a* = 0.50 for population factors). Furthermore, one could introduce secondary loadings in addition to the main loadings. These could be modeled to maintain orthogonality (e.g., with alternating signs) or to produce obliqueness in the population model. This would be particularly interesting because in most cases, obliqueness is more likely than orthogonality despite the popularity of Varimax (Fabrigar et al., 1999; Browne, 2001). For different conditions of obliqueness, GPR could be investigated for oblique rotation with Promax, Oblimin/Quartimin, or also Geomin as a newer method for oblique and orthogonal rotation (Browne, 2001; Hattori et al., 2017).

The GPR algorithm for the oblique rotation criteria Geomin and Oblimin was also used by Dien (2010), who compared different rotation methods in PCA on event-related potentials (ERP) from simulated EEG data. He found less favorable results for Geomin and Oblimin, but he could not differentiate whether the effect was due to the rotation criteria or due to the GPR-algorithm used to optimize them. One could build on these findings to examine whether GPR performs better or worse for the rotation of ERPs when compared to other rotation approaches. This should be conducted for Varimax rotation, for which misallocation of variance is a known problem in ERP data (Wood and McCarthy, 1984; Beauducel and Debener, 2003). It could be compared whether GPR-based Varimax rotation produces more or less misallocation of variance than Varimax rotation by the built-in SPSS procedure.

### Conclusion and Recommendations

To conclude, our study supports that Varimax rotation by GPR, a rotation algorithm that is openly available and usable in free software like R (Bernaards and Jennrich, 2005), is a feasible alternative to the built-in Varimax procedure in the commercial software package SPSS. Both the simulation study and the empirical example show that results of GPR-Varimax and SPSS-Varimax are comparable. In the simulation, the few and very small differences in rotation performance in favor of SPSS were fully eliminated when loadings were Kaiser normalized before rotation and 250 iterations were allowed per rotation. This was true for both the single-optimum and the double-optimum (global and local) model of simple structure in the population. In the double-optimum case, GPR-Varimax performed even better than SPSS-Varimax when loadings were Kaiser normalized and the best solution from multiple random start loadings was selected. When a clear simple structure cannot be expected, it can be advantageous to test 10 random start loading matrices and select the solution with the maximum Varimax criterion. Combining the results from the simulation study, we recommend using Kaiser normalized loadings and a minimum of 250 iterations per rotation when GPR-Varimax is conducted. Furthermore, users of GPR-Varimax should insert the unrotated loading matrix and an additional 10 random loading matrices as start loadings and select the solution with the maximum Varimax criterion. We provide the code for conducting GPR-Varimax with the recommended adjustments and selection of the best solution in R and SPSS MATRIX language in the Supplementary Materials of this paper.

## Author Contributions

All authors listed have made a substantial, direct and intellectual contribution to the work, and approved it for publication.

## Conflict of Interest Statement

The authors declare that the research was conducted in the absence of any commercial or financial relationships that could be construed as a potential conflict of interest.

## Footnotes

1 http://www.stat.ucla.edu/research/gpa
