# Supplementary material for: Varimax Rotation Based on Gradient Projection Is a Feasible Alternative to SPSS
Source: Front Psychol. 2019 Mar 26;10:645. doi: 10.3389/fpsyg.2019.00645 (PMC6443893; doi:10.3389/fpsyg.2019.00645)
Supplement: Supplementary file 2 [file Data_Sheet_1.PDF]

Supplementary Material

Varimax rotation based on gradient projection is a feasible alternative to SPSS

Anneke Cleopatra Weide*, **André Beauducel**

*** Correspondence:** Anneke Cleopatra Weide: aweide@uni-bonn.de

1 Supplementary Code for GPR-Varimax

The following code follows the suggestions we give in the manuscript for optimal results of GPR-Varimax.

1.1 Code for GPR-Varimax in R

```
################################
#### EXAMPLE FOR GPR-VARIMAX

#### WITH KAISER-NORMALIZATION
#### SELECTION OF SOLUTION WITH GREATEST VARIMAX FROM UNROTATED LOADINGS AND
10 RANDOM START LOADINGS

#### additions to code for GPArotation (Bernaards & Jennrich, 2005):
#### Kaiser-normalization of loadings before rotation
#### loops for selecting best solution from identity-T and 10 random Ts


setwd ("F:/Gradient projection rotation/Data/R codes and data")

install.packages("GPArotation")
library("GPArotation")

library(foreign)

### settings:

n_fac <- 3
n_var <- 18
n_random <-10
n_iter <- 250

### get unrotated loading matrix A:

A <- as.matrix(read.table("A_norot_Sample1_3C.txt", sep = ";"))
```

```
### Kaiser-normalization: divide loadings by square root of
communalities

A_sq <-A^2
H_sq <- rowSums(A_sq, na.rm = FALSE, dims = 1)
H_root <- H_sq^0.5
H_diag <- diag(H_root)

A_kais <- solve(H_diag)%*%A
A <- A_kais

###  GPR-Varimax
### first use identity-T (unrotated start loadings)

GPR_ident <- GPForth(A, Tmat=diag(ncol(A)), normalize=FALSE, eps=1e-5,
maxit=n_iter,
        method="varimax", methodArgs=NULL)

A_sol1 <- GPR_ident[["loadings"]]

### reverse Kaiser-normalization after rotation:

A_sol1 <- diag(H_root)%*%A_sol1

### Varimax-criterion for unrotated start loadings (T = identity)

A_sq <- A_sol1^2
mn_A_sq1 <- colSums(A_sq)/n_var
mn_A_sq2 <- matrix(rep(mn_A_sq1,each=n_var), nrow=n_var, byrow=FALSE)

A_diff <- A_sq - mn_A_sq2
A_diff_sq <- A_diff^2
A_diff_cssq <- colSums(A_diff_sq, na.rm = FALSE, dims = 1)

var_A_sq <- A_diff_cssq/n_var
var_A_rssq <- sum(var_A_sq)
vari_1 <- var_A_rssq/n_fac

### loop across 10 random start loadings and replace solution if Varimax
criterion gets larger
### set preliminary varicrit and A solution to solution from T=identity:

A_sol <- A_sol1

vari_crit <- vari_1


for  (i_random in 1:n_random){

  Random.Start <- function(n_fac){
    qr.Q(qr(matrix(rnorm(n_fac*n_fac),n_fac)))
  }

  T_rnd <- Random.Start(n_fac)
```

```
  GPR_rand <- GPForth(A, Tmat=T_rnd, normalize=FALSE, eps=1e-5, maxit=n_iter,
                     method="varimax", methodArgs=NULL)

  A_solr <- GPR_rand[["loadings"]]

### reverse Kaiser-normalization after rotation:

A_solr <- diag(H_root)%*%A_solr

### Varimax criterion for each solution from random start loadings:

A_sq <- A_solr^2
mn_A_sq1 <- colSums(A_sq)/n_var
mn_A_sq2 <- matrix(rep(mn_A_sq1,each=n_var), nrow=n_var, byrow=FALSE)

A_diff <- A_sq - mn_A_sq2
A_diff_sq <- A_diff^2
A_diff_cssq <- colSums(A_diff_sq, na.rm = FALSE, dims = 1)

var_A_sq <- A_diff_cssq/n_var
var_A_rssq <- sum(var_A_sq)
vari_rnd <- var_A_rssq/n_fac


### select solution with largest Varimax criterion:

if(vari_rnd > vari_crit){
  vari_crit <- vari_rnd
  A_sol <- A_solr
}

}

View(A_sol1)
View(A_sol)
View(vari_crit)


write.table (A_sol, "A_GPR_R_Sample1_3C.txt", sep = ";")
```

1.2 Code for GPR-Varimax in SPSS Matrix

```
*****************************************************************************
***************
**** EXAMPLE FOR GPR-VARIMAX


**** WITH KAISER-NORMALIZATION

**** SELECTION OF SOLUTION WITH GREATEST VARIMAX
**** FROM UNROTATED LOADINGS AND 10 RANDOM START LOADINGS
*****************************************************************************
****************


*** changes to code from http://www.stat.ucla.edu/research/gpa/orthogonal.SPS
(Bernaards & Jennrich, 2005):

***  Kaiser-normalization of loadings before rotation
***  loops for selecting best solution from identity-T and 10 random Ts

*** Tmat for identity as Tmat = ident(n_fac) instead of Tmat={1, 0; 0, 1}
*** #iter = 0 to n_iter
*** addition under compute X=Tmat-al*Gp.: compute X=TRUNC(1000000*X)&/1000000.
*** (last step ensures a symmetric matrix for SVD of X)


set mxloop = 100000000 mtindex = 500000.

*****************************************************.
*****************************************************.
*  VARIMAX.
*****************************************************.
*****************************************************.

MATRIX.

    *settings:

    compute n_fac = 3.
    compute n_var = 18.
    compute n_random = 10.
    compute n_iter = 250.

    * get unrotated loading matrix A:

    get A /file = "F:\Gradient projection rotation\Data\SPSS data\Sample
    1\A_norot_Sample1_3C.sav".

    * Kaiser normalization: divide loadings by square root of communalities:

    compute H_sq = rssq(A).
    compute H_root =  (H_sq)&**0.5.
    compute A_kais=INV(Mdiag(H_root))*A.
```

```
* GPR-Varimax:

**********************************************************
    *** first use identity-T (unrotated start loadings)

compute A = A_kais.
compute Tmat = ident(n_fac).
compute al=1.
compute L=A * Tmat.
* .
* Varimax.
* .
compute L2 = L&*L.
compute n  = nrow(L2).
compute cm = make(n,n,1)/n.
compute QL = L2 - cm*L2.
compute ft = -trace(T(QL)*QL)/4.
compute Gq = -L&*QL.
* .
* end Varimax.
* .
compute f=ft.
compute G=T(A)*Gq.
loop #iter=0 to n_iter .
    compute M=T(Tmat) * G.
    compute S=(M + T(M))/2.
    compute Gp=G - Tmat*S.
    compute s=sqrt(trace(T(Gp) * Gp)).
    compute sl = lg10(s).
    compute outp ={#iter, ft, sl, al}.
    *print outp.
    do if (s < 0.00001).
        break.
    end if.
    compute al=2*al.
    loop #i = 0 to 10.
        compute X=Tmat-al*Gp.
        compute X=TRUNC(1000000*X)&/1000000.
        call SVD (X,u,d,v).
        compute Tt=U*T(V).
        compute L=A*Tt.
        * .
        * Varimax.
        * .
        compute L2 = L&*L.
        compute n  = nrow(L2).
        compute cm = make(n,n,1)/n.
        compute QL = L2 - cm*L2.
        compute ft = -trace(T(QL)*QL)/4.
        compute Gq = -L&*QL.
        * .
        * end Varimax.
        * .
        do if (ft < f-.5*s*s*al).
```

```
            compute i=10.
        end if.
        do if (ft > f-.5*s*s*al).
            compute al=al/2.
        end if.
    end loop.
    compute Tmat=Tt.
    compute f=ft.
    compute G=T(A)*Gq.
end loop.
compute A_sol1=A*Tmat.
*print A_sol1.
*print Tmat.

*reverse Kaiser-normalization after rotation:

compute A_sol1= mdiag(H_root)*A_sol1.


*************************************************************************
    ** Varimax-criterion for unrotated start loadings (T = identity)

compute A_sq = A_sol1&**2.
compute mn_A_sq1 = csum(A_sq)/n_var.

loop i_fac = 1 to n_fac.
    compute mn_multi = make(n_var,1,mn_A_sq1(1,i_fac)).
    do if i_fac = 1.
        compute mn_A_sq2 = mn_multi.
    end if.
    do if i_fac > 1.
        compute mn_A_sq2 = {mn_A_sq2, mn_multi}.
    end if.
end loop.

compute A_diff = A_sq - mn_A_sq2.
compute var_A_sq = cssq(A_diff)/n_var.
compute vari_1 = rsum(var_A_sq)/n_fac.



*****************************************************************************
****************************
        ** loop across 10 random start loadings and replace solution if
Varimax criterion gets larger

    *set preliminary varicrit and A solution to solution from T=identity:

    compute A_sol = A_sol1.
    compute varicrit = vari_1.

    loop i_random=1 to n_random.

        compute A = A_kais.
        compute TRA = UNIFORM(n_fac, n_fac).
```

```
compute Tmat = GSCH(TRA).
compute al=1.
compute L=A * Tmat.
* .
* Varimax.
* .
compute L2 = L&*L.
compute n  = nrow(L2).
compute cm = make(n,n,1)/n.
compute QL = L2 - cm*L2.
compute ft = -trace(T(QL)*QL)/4.
compute Gq = -L&*QL.
* .
* end Varimax.
* .
compute f=ft.
compute G=T(A)*Gq.
loop #iter=0 to n_iter .
    compute M=T(Tmat) * G.
    compute S=(M + T(M))/2.
    compute Gp=G - Tmat*S.
    compute s=sqrt(trace(T(Gp) * Gp)).
    compute sl = lg10(s).
    compute outp ={#iter, ft, sl, al}.
    *print outp.
    do if (s < 0.00001).
        break.
    end if.
    compute al=2*al.
    loop #i = 0 to 10.
        compute X=Tmat-al*Gp.
        compute X=TRUNC(1000000*X)&/1000000.
        call SVD (X,u,d,v).
        compute Tt=U*T(V).
        compute L=A*Tt.
        * .
        * Varimax.
        * .
        compute L2 = L&*L.
        compute n  = nrow(L2).
        compute cm = make(n,n,1)/n.
        compute QL = L2 - cm*L2.
        compute ft = -trace(T(QL)*QL)/4.
        compute Gq = -L&*QL.
        * .
        * end Varimax.
        * .
        do if (ft < f-.5*s*s*al).
            compute i=10.
        end if.
        do if (ft > f-.5*s*s*al).
            compute al=al/2.
        end if.
    end loop.
    compute Tmat=Tt.
    compute f=ft.
```

```
            compute G=T(A)*Gq.
        end loop.
        compute A_solr=A*Tmat.
        *print A_solr.
        *print Tmat.

        *reverse Kaiser-normalization after rotation:

        compute A_solr= mdiag(H_root)*A_solr.


        **** Varimax criterion for each solution from random start loadings:

        compute A_sq = A_solr&**2.
        compute mn_A_sq1 = csum(A_sq)/n_var.

        loop i_fac = 1 to n_fac.
            compute mn_multi = make(n_var,1,mn_A_sq1(1,i_fac)).
            do if i_fac = 1.
                compute mn_A_sq2 = mn_multi.
            end if.
            do if i_fac > 1.
                compute mn_A_sq2 = {mn_A_sq2, mn_multi}.
            end if.
        end loop.

        compute A_diff = A_sq - mn_A_sq2.
        compute var_A_sq = cssq(A_diff)/n_var.
        compute vari_rnd = rsum(var_A_sq)/n_fac.

        * select solution with largest Varimax criterion:

        do if  vari_rnd > varicrit.
            compute A_sol = A_solr.
            compute varicrit = vari_rnd.
        end if.

    end loop.

    print A_sol1.
    print A_sol.
    print varicrit.

save A_sol /outfile = "F:\Gradient projection rotation\Data\SPSS data\Sample
1\A_GPR_SPSSMatr_Sample1_3C.sav".


END MATRIX.
```

2 Supplementary Tables

Supplementary Table 1

Exemplary loading matrix obtained from GPR-Varimax implemented in SPSS Matrix and R for Sample 1 from the simulation and rotation of 3 components with 18 variables, n = 100, and single-optimum simple structure in the population

	Unrotated Loadings			GPR-Varimax solution from SPSS Matrix			GPR-Varimax solution from R		
	C1	C2	C3	C1	C2	C3	C1	C2	C3
V1	.56	-.16	.17	.59	-.03	.13	.59	-.03	.13
V2	.61	-.46	.01	.74	-.02	-.18	.74	-.02	-.18
V3	.60	-.36	.02	.69	-.05	-.11	.69	-.05	-.11
V4	.56	.02	-.15	.45	-.36	.05	.45	-.36	.05
V5	.47	-.35	.15	.59	.09	-.05	.59	.09	-.05
V6	.48	-.06	.12	.47	-.09	.14	.47	-.09	.14
V7	-.33	-.40	.41	-.04	.65	-.11	-.04	.65	-.11
V8	.15	-.41	.43	.40	.47	.00	.40	.47	.00
V9	-.29	-.01	.63	-.13	.60	.33	-.13	.60	.33
V10	-.28	-.31	.41	-.02	.59	-.03	-.02	.59	-.03
V11	-.27	-.33	.31	-.03	.51	-.11	-.03	.51	-.11
V12	-.30	-.34	.34	-.04	.56	-.10	-.04	.56	-.10
V13	.14	.52	.34	-.04	-.07	.63	-.04	-.07	.63
V14	.08	.46	.38	-.06	.02	.60	-.06	.02	.60
V15	.09	.38	.27	-.04	-.03	.47	-.04	-.03	.47
V16	.41	.25	.32	.31	-.06	.49	.31	-.06	.49
V17	.06	.45	.24	-.10	-.08	.50	-.10	-.08	.50
V18	.37	.36	.42	.24	-.03	.62	.24	-.03	.62

Note. GPR = gradient-projection based rotation. GPR-Varimax was conducted with the suggested settings: Kaiser-normalized loadings, 250 iterations per rotation, and selection of the best solution from the unrotated loading matrix and an additional 10 multiple random start loading matrices.

Supplementary Table 2

Varimax criterion for component rotation by SPSS-Varimax and GPR-Varimax for the single-optimum model and 4 variables per component – Means and standard deviations of 1,000 samples

			25 iterations		250 iterations	
k	N	Rotation method	No Kaiser	With Kaiser	No Kaiser	With Kaiser
3	100	SPSS	.0408 (.00494)	.0406 (.00501)	.0408 (.00494)	.0406 (.00501)
		GPR-unrotated	.0408 (.00494)	.0406 (.00502)	.0408 (.00494)	.0406 (.00502)
		GPR-random 1	.0408 (.00494)	.0406 (.00502)	.0408 (.00494)	.0406 (.00502)
		GPR-random 10	.0408 (.00494)	.0406 (.00500)	.0408 (.00494)	.0406 (.00501)
	300	SPSS	.0417 (.00269)	.0417 (.00270)	.0417 (.00269)	.0417 (.00270)
		GPR-unrotated	.0417 (.00269)	.0417 (.00270)	.0417 (.00269)	.0417 (.00270)
		GPR-random 1	.0417 (.00269)	.0417 (.00270)	.0417 (.00269)	.0417 (.00270)
		GPR-random 10	.0417 (.00269)	.0417 (.00270)	.0417 (.00269)	.0417 (.00270)
6	100	SPSS	.0226 (.00237)	.0223 (.00244)	.0226 (.00237)	.0223 (.00244)
		GPR-unrotated	.0226 (.00237)	.0223 (.00244)	.0226 (.00237)	.0223 (.00244)
		GPR-random 1	.0226 (.00237)	.0223 (.00246)	.0226 (.00237)	.0223 (.00245)
		GPR-random 10	.0226 (.00237)	.0223 (.00244)	.0226 (.00237)	.0223 (.00245)
	300	SPSS	.0249 (.00134)	.0249 (.00134)	.0249 (.00134)	.0240 (.00134)
		GPR-unrotated	.0249 (.00134)	.0249 (.00134)	.0249 (.00134)	.0240 (.00134)
		GPR-random 1	.0249 (.00134)	.0249 (.00134)	.0249 (.00134)	.0240 (.00134)
		GPR-random 10	.0249 (.00134)	.0249 (.00134)	.0249 (.00134)	.0240 (.00134)
9	100	SPSS	.0147 (.00125)	.0145 (.00130)	.0147 (.00125)	.0145 (.00129)
		GPR-unrotated	.0147 (.00125)	.0145 (.00130)	.0147 (.00125)	.0145 (.00129)
		GPR-random 1	.0147 (.00125)	.0145 (.00130)	.0147 (.00125)	.0145 (.00129)
		GPR-random 10	.0147 (.00125)	.0145 (.00130)	.0147 (.00125)	.0145 (.00129)
	300	SPSS	.0170 (.00084)	.0170 (.00085)	.0170 (.00084)	.0170 (.00085)
		GPR-unrotated	.0170 (.00084)	.0170 (.00085)	.0170 (.00084)	.0170 (.00085)
		GPR-random 1	.0170 (.00084)	.0170 (.00085)	.0170 (.00084)	.0170 (.00085)
		GPR-random 10	.0170 (.00084)	.0170 (.0085)	.0170 (.00084)	.0170 (.00085)
12	100	SPSS	.0108 (.00077)	.0107 (.00079)	.0108 (.00076)	.0107 (.00079)
		GPR-unrotated	.0108 (.00077)	.0106 (.00080)	.0108 (.00076)	.0107 (.00080)
		GPR-random 1	.0108 (.00077)	.0106 (.00080)	.0108 (.00076)	.0107 (.00080)
		GPR-random 10	.0108 (.00077)	.0106 (.00080)	.0108 (.00076)	.0107 (.00080)
	300	SPSS	.0126 (.00057)	.0125 (.00057)	.0126 (.00057)	.0125 (.00057)
		GPR-unrotated	.0126 (.00057)	.0125 (.00057)	.0126 (.00057)	.0125 (.00057)
		GPR-random 1	.0126 (.00057)	.0125 (.00057)	.0126 (.00057)	.0125 (.00057)
		GPR-random 10	.0126 (.00057)	.0125 (.00057)	.0126 (.00057)	.0125 (.00057)

Note. GPR = gradient-projection based rotation. *k* = number of components. Rotation after principal component analysis of simulated data.

Supplementary Table 3

Mean congruence with population components for component rotation by SPSS-Varimax and GPR-Varimax for the single-optimum model and 4 variables per component – Means and standard deviations of 1,000 samples

			25 iterations		250 iterations	
k	N	Rotation method	No Kaiser	With Kaiser	No Kaiser	With Kaiser
3	100	SPSS	.944 (.0282)	.944 (.0291)	.944 (.0282)	.944 (.0291)
		GPR-unrotated	.944 (.0282)	.944 (.0296)	.944 (.0282)	.944 (.0296)
		GPR-random 1	.944 (.0282)	.944 (.0297)	.944 (.0282)	.944 (.0295)
		GPR-random 10	.944 (.0282)	.944 (.0291)	.944 (.0282)	.944 (.0291)
	300	SPSS	.983 (.0052)	.983 (.0052)	.983 (.0052)	.983 (.0052)
		GPR-unrotated	.983 (.0052)	.983 (.0052)	.983 (.0052)	.983 (.0052)
		GPR-random 1	.983 (.0052)	.983 (.0052)	.983 (.0052)	.983 (.0052)
		GPR-random 10	.983 (.0052)	.983 (.0052)	.983 (.0052)	.983 (.0052)
6	100	SPSS	.862 (.0453)	.863 (.0462)	.862 (.0453)	.863 (.0461)
		GPR-unrotated	.862 (.0453)	.863 (.0463)	.862 (.0452)	.863 (.0461)
		GPR-random 1	.862 (.0453)	.863 (.0462)	.862 (.0452)	.863 (.0460)
		GPR-random 10	.862 (.0453)	.863 (.0457)	.862 (.0452)	.863 (.0461)
	300	SPSS	.962 (.0064)	.962 (.0064)	.962 (.0064)	.962 (.0064)
		GPR-unrotated	.962 (.0064)	.962 (.0064)	.962 (.0064)	.962 (.0064)
		GPR-random 1	.962 (.0064)	.962 (.0064)	.962 (.0064)	.962 (.0064)
		GPR-random 10	.962 (.0064)	.962 (.0064)	.962 (.0064)	.962 (.0064)
9	100	SPSS	.786 (.0452)	.789 (.0452)	.786 (.0452)	.789 (.0451)
		GPR-unrotated	.785 (.0460)	.788 (.0458)	.785 (.0457)	.789 (.0452)
		GPR-random 1	.785 (.0460)	.788 (.0458)	.785 (.0457)	.789 (.0452)
		GPR-random 10	.785 (.0460)	.788 (.0458)	.785 (.0457)	.789 (.0452)
	300	SPSS	.940 (.0075)	.941 (.0073)	.940 (.0075)	.941 (.0073)
		GPR-unrotated	.940 (.0074)	.941 (.0073)	.940 (.0074)	.941 (.0073)
		GPR-random 1	.940 (.0075)	.941 (.0073)	.940 (.0075)	.941 (.0073)
		GPR-random 10	.940 (.0075)	.941 (.0073)	.940 (.0075)	.941 (.0073)
12	100	SPSS	.729 (.0412)	.732 (.0405)	.729 (.0411)	.732 (.0406)
		GPR-unrotated	.727 (.0416)	.731 (.0411)	.729 (.0410)	.732 (.0410)
		GPR-random 1	.727 (.0416)	.731 (.0411)	.729 (.0410)	.732 (.0410)
		GPR-random 10	.727 (.0416)	.731 (.0411)	.729 (.0410)	.732 (.0410)
	300	SPSS	.918 (.0088)	.918 (.0086)	.918 (.0088)	.918 (.0086)
		GPR-unrotated	.918 (.0088)	.918 (.0086)	.918 (.0088)	.918 (.0086)
		GPR-random 1	.918 (.0088)	.918 (.0086)	.918 (.0088)	.918 (.0086)
		GPR-random 10	.918 (.0088)	.918 (.0086)	.918 (.0088)	.918 (.0086)

Note. GPR = gradient-projection based rotation. *k* = number of components. Rotation after principal component analysis of simulated data.

Supplementary Table 4

RMSE as deviation from population components for rotation by SPSS-Varimax and GPR-Varimax for the single-optimum model and 4 variables per component – Means and standard deviations of 1,000 samples

			25 iterations		250 iterations	
k	N	Rotation method	No Kaiser	With Kaiser	No Kaiser	With Kaiser
3	100	SPSS	.128 (.0295)	.128 (.0314)	.128 (.0295)	.128 (.0314)
		GPR-unrotated	.128 (.0295)	.128 (.0317)	.128 (.0295)	.128 (.0317)
		GPR-random 1	.128 (.0295)	.128 (.0317)	.128 (.0295)	.128 (.0316)
		GPR-random 10	.128 (.0295)	.128 (.0300)	.128 (.0295)	.128 (.0314)
	300	SPSS	.070 (.0105)	.070 (.0104)	.070 (.0105)	.070 (.0104)
		GPR-unrotated	.070 (.0105)	.070 (.0104)	.070 (.0105)	.070 (.0104)
		GPR-random 1	.070 (.0105)	.070 (.0104)	.070 (.0105)	.070 (.0104)
		GPR-random 10	.070 (.0105)	.070 (.0104)	.070 (.0105)	.070 (.0104)
6	100	SPSS	.149 (.0321)	.148 (.0322)	.149 (.0321)	.148 (.0322)
		GPR-unrotated	.149 (.0321)	.149 (.0327)	.149 (.0321)	.148 (.0323)
		GPR-random 1	.149 (.0321)	.148 (.0325)	.149 (.0321)	.148 (.0320)
		GPR-random 10	.149 (.0321)	.148 (.0318)	.149 (.0321)	.148 (.0318)
	300	SPSS	.076 (.0061)	.076 (.0061)	.076 (.0061)	.076 (.0060)
		GPR-unrotated	.076 (.0061)	.076 (.0061)	.076 (.0061)	.076 (.0060)
		GPR-random 1	.076 (.0061)	.076 (.0061)	.076 (.0061)	.076 (.0060)
		GPR-random 10	.076 (.0061)	.076 (.0061)	.076 (.0061)	.076 (.0060)
9	100	SPSS	.158 (.0246)	.157 (.0250)	.158 (.0246)	.157 (.0250)
		GPR-unrotated	.158 (.0250)	.158 (.0254)	.158 (.0251)	.157 (.0252)
		GPR-random 1	.158 (.0250)	.158 (.0254)	.158 (.0251)	.157 (.0252)
		GPR-random 10	.158 (.0250)	.158 (.0254)	.158 (.0251)	.157 (.0252)
	300	SPSS	.078 (.0045)	.077 (.0045)	.078 (.0045)	.077 (.0045)
		GPR-unrotated	.078 (.0045)	.077 (.0045)	.078 (.0045)	.077 (.0045)
		GPR-random 1	.078 (.0045)	.077 (.0045)	.078 (.0045)	.077 (.0045)
		GPR-random 10	.078 (.0045)	.077 (.0045)	.078 (.0045)	.077 (.0045)
12	100	SPSS	.159 (.0191)	.158 (.0193)	.159 (.0190)	.158 (.0193)
		GPR-unrotated	.160 (.0194)	.159 (.0199)	.159 (.0189)	.159 (.0193)
		GPR-random 1	.160 (.0194)	.159 (.0199)	.159 (.0189)	.159 (.0193)
		GPR-random 10	.160 (.0194)	.159 (.0199)	.159 (.0189)	.159 (.0193)
	300	SPSS	.080 (.0039)	.079 (.0039)	.080 (.0039)	.079 (.0039)
		GPR-unrotated	.080 (.0040)	.079 (.0039)	.080 (.0040)	.079 (.0039)
		GPR-random 1	.080 (.0039)	.079 (.0039)	.080 (.0039)	.079 (.0039)
		GPR-random 10	.080 (.0039)	.079 (.0039)	.080 (.0039)	.079 (.0039)

Note. GPR = gradient-projection based rotation. *k* = number of components. Rotation after principal component analysis of simulated data.

Supplementary Table 5

Varimax criterion for component rotation by SPSS-Varimax and GPR-Varimax for the single-optimum model and 6 variables per component – Means and standard deviations of 1,000 samples

			25 iterations		250 iterations	
k	N	Rotation method	No Kaiser	With Kaiser	No Kaiser	With Kaiser
3	100	SPSS	.0311 (.00360)	.0310 (.00362)	.0311 (.00360)	.0310 (.00362)
		GPR-unrotated	.0311 (.00360)	.0310 (.00362)	.0311 (.00360)	.0310 (.00362)
		GPR-random 1	.0311 (.00360)	.0310 (.00362)	.0311 (.00360)	.0310 (.00362)
		GPR-random 10	.0311 (.00360)	.0310 (.00362)	.0311 (.00360)	.0310 (.00362)
	300	SPSS	.0311 (.00205)	.0311 (.00205)	.0311 (.00205)	.0311 (.00205)
		GPR-unrotated	.0311 (.00205)	.0311 (.00205)	.0311 (.00205)	.0311 (.00205)
		GPR-random 1	.0311 (.00205)	.0311 (.00205)	.0311 (.00205)	.0311 (.00205)
		GPR-random 10	.0311 (.00205)	.0311 (.00205)	.0311 (.00205)	.0311 (.00205)
6	100	SPSS	.0176 (.00174)	.0175 (.00176)	.0176 (.00174)	.0175 (.00176)
		GPR-unrotated	.0176 (.00174)	.0175 (.00176)	.0176 (.00174)	.0175 (.00176)
		GPR-random 1	.0176 (.00175)	.0175 (.00177)	.0176 (.00175)	.0175 (.00177)
		GPR-random 10	.0176 (.00175)	.0175 (.00177)	.0176 (.00175)	.0175 (.00177)
	300	SPSS	.0187 (.00095)	.0187 (.00095)	.0187 (.00095)	.0187 (.00095)
		GPR-unrotated	.0187 (.00095)	.0187 (.00095)	.0187 (.00095)	.0187 (.00095)
		GPR-random 1	.0187 (.00095)	.0187 (.00095)	.0187 (.00095)	.0187 (.00095)
		GPR-random 10	.0187 (.00095)	.0187 (.00095)	.0187 (.00095)	.0187 (.00095)
9	100	SPSS	.0114 (.00103)	.0113 (.00104)	.0114 (.00103)	.0113 (.00104)
		GPR-unrotated	.0114 (.00103)	.0113 (.00105)	.0114 (.00103)	.0113 (.00105)
		GPR-random 1	.0114 (.00103)	.0113 (.00105)	.0113 (.00105)	.0112 (.00107)
		GPR-random 10	.0114 (.00103)	.0113 (.00105)	.0113 (.00105)	.0112 (.00107)
	300	SPSS	.0129 (.00059)	.0129 (.00059)	.0129 (.00059)	.0129 (.00059)
		GPR-unrotated	.0129 (.00059)	.0129 (.00059)	.0129 (.00059)	.0129 (.00059)
		GPR-random 1	.0129 (.00059)	.0129 (.00059)	.0129 (.00059)	.0129 (.00059)
		GPR-random 10	.0129 (.00059)	.0129 (.00059)	.0129 (.00059)	.0129 (.00059)
12	100	SPSS	.0083 (.00062)	.0082 (.00065)	.0083 (.00062)	.0082 (.00065)
		GPR-unrotated	.0083 (.00063)	.0082 (.00064)	.0083 (.00063)	.0082 (.00064)
		GPR-random 1	.0083 (.00063)	.0082 (.00064)	.0083 (.00063)	.0082 (.00064)
		GPR-random 10	.0083 (.00063)	.0082 (.00064)	.0083 (.00063)	.0082 (.00064)
	300	SPSS	.0097 (.00039)	.0096 (.00039)	.0097 (.00039)	.0096 (.00039)
		GPR-unrotated	.0097 (.00039)	.0096 (.00039)	.0097 (.00039)	.0096 (.00039)
		GPR-random 1	.0097 (.00039)	.0096 (.00039)	.0097 (.00039)	.0096 (.00039)
		GPR-random 10	.0097 (.00039)	.0096 (.00039)	.0097 (.00039)	.0096 (.00039)

Note. GPR = gradient-projection based rotation. *k* = number of components. Rotation after principal component analysis of simulated data.

Supplementary Table 6

Mean congruence with population components for component rotation by SPSS-Varimax and GPR-Varimax for the single-optimum model and 6 variables per component – Means and standard deviations of 1,000 samples

			25 iterations		250 iterations	
k	N	Rotation method	No Kaiser	With Kaiser	No Kaiser	With Kaiser
3	100	SPSS	.949 (.0160)	.949 (.0159)	.949 (.0160)	.949 (.0159)
		GPR-unrotated	.949 (.0160)	.949 (.0158)	.949 (.0160)	.949 (.0159)
		GPR-random 1	.949 (.0160)	.949 (.0159)	.949 (.0160)	.949 (.0159)
		GPR-random 10	.949 (.0160)	.949 (.0159)	.949 (.0160)	.949 (.0159)
	300	SPSS	.984 (.0039)	.984 (.0039)	.984 (.0039)	.984 (.0039)
		GPR-unrotated	.984 (.0039)	.984 (.0039)	.984 (.0039)	.984 (.0039)
		GPR-random 1	.984 (.0039)	.984 (.0039)	.984 (.0039)	.984 (.0039)
		GPR-random 10	.984 (.0039)	.984 (.0039)	.984 (.0039)	.984 (.0039)
6	100	SPSS	.883 (.0261)	.884 (.0255)	.883 (.0261)	.884 (.0255)
		GPR-unrotated	.882 (.0263)	.884 (.0255)	.883 (.0261)	.884 (.0255)
		GPR-random 1	.883 (.0262)	.884 (.0255)	.883 (.0261)	.884 (.0255)
		GPR-random 10	.883 (.0261)	.884 (.0255)	.883 (.0261)	.884 (.0255)
	300	SPSS	.964 (.0044)	.964 (.0043)	.964 (.0043)	.964 (.0043)
		GPR-unrotated	.964 (.0044)	.964 (.0043)	.964 (.0043)	.964 (.0043)
		GPR-random 1	.964 (.0044)	.964 (.0043)	.964 (.0043)	.964 (.0043)
		GPR-random 10	.964 (.0044)	.964 (.0043)	.964 (.0043)	.964 (.0043)
9	100	SPSS	.811 (.0350)	.814 (.0343)	.811 (.0350)	.814 (.0343)
		GPR-unrotated	.811 (.0355)	.814 (.0345)	.811 (.0350)	.814 (.0341)
		GPR-random 1	.811 (.0355)	.814 (.0345)	.811 (.0350)	.814 (.0341)
		GPR-random 10	.811 (.0355)	.814 (.0345)	.811 (.0350)	.814 (.0341)
	300	SPSS	.944 (.0050)	.944 (.0050)	.944 (.0050)	.944 (.0050)
		GPR-unrotated	.944 (.0053)	.944 (.0050)	.944 (.0053)	.944 (.0050)
		GPR-random 1	.944 (.0050)	.944 (.0050)	.944 (.0050)	.944 (.0050)
		GPR-random 10	.944 (.0050)	.944 (.0050)	.944 (.0050)	.944 (.0050)
12	100	SPSS	.755 (.0324)	.758 (.0321)	.755 (.0324)	.758 (.0321)
		GPR-unrotated	.754 (.0334)	.757 (.0329)	.755 (.0326)	.758 (.0322)
		GPR-random 1	.754 (.0334)	.757 (.0329)	.755 (.0326)	.758 (.0322)
		GPR-random 10	.754 (.0334)	.757 (.0329)	.755 (.0326)	.758 (.0322)
	300	SPSS	.924 (.0054)	.925 (.0053)	.9244 (.0054)	.925 (.0053)
		GPR-unrotated	.924 (.0054)	.925 (.0053)	.924 (.0054)	.925 (.0053)
		GPR-random 1	.924 (.0054)	.925 (.0053)	.924 (.0054)	.925 (.0053)
		GPR-random 10	.924 (.0054)	.925 (.0053)	.924 (.0054)	.925 (.0053)

Note. GPR = gradient-projection based rotation. *k* = number of components. Rotation after principal component analysis of simulated data.

Supplementary Table 7

RMSE as deviation from population components for rotation by SPSS-Varimax and GPR-Varimax for the single-optimum model and 6 variables per component – Means and standard deviations of 1,000 samples

			25 iterations		250 iterations	
k	N	Rotation method	No Kaiser	With Kaiser	No Kaiser	With Kaiser
3	100	SPSS	.115 (.0159)	.115 (.0158)	.115 (.0159)	.115 (.0158)
		GPR-unrotated	.115 (.0159)	.115 (.0158)	.115 (.0159)	.115 (.0158)
		GPR-random 1	.115 (.0159)	.115 (.0158)	.115 (.0159)	.115 (.0158)
		GPR-random 10	.115 (.0159)	.115 (.0158)	.115 (.0159)	.115 (.0158)
	300	SPSS	.065 (.0073)	.064 (.0073)	.065 (.0073)	.064 (.0073)
		GPR-unrotated	.065 (.0073)	.064 (.0073)	.065 (.0073)	.064 (.0073)
		GPR-random 1	.065 (.0073)	.064 (.0073)	.065 (.0073)	.064 (.0073)
		GPR-random 10	.065 (.0073)	.064 (.0073)	.065 (.0073)	.064 (.0073)
6	100	SPSS	.126 (.0151)	.126 (.0145)	.126 (.0151)	.126 (.0145)
		GPR-unrotated	.126 (.0154)	.126 (.0149)	.126 (.0151)	.126 (.0145)
		GPR-random 1	.126 (.0151)	.126 (.0145)	.126 (.0151)	.126 (.0145)
		GPR-random 10	.126 (.0151)	.126 (.0145)	.126 (.0151)	.126 (.0145)
	300	SPSS	.069 (.0034)	.068 (.0038)	.069 (.0039)	.068 (.0038)
		GPR-unrotated	.069 (.0034)	.068 (.0038)	.069 (.0039)	.068 (.0038)
		GPR-random 1	.069 (.0034)	.068 (.0038)	.069 (.0039)	.068 (.0038)
		GPR-random 10	.069 (.0034)	.068 (.0038)	.069 (.0039)	.068 (.0038)
9	100	SPSS	.135 (.0173)	.134 (.0169)	.135 (.0173)	.134 (.0169)
		GPR-unrotated	.136 (.0178)	.134 (.0172)	.135 (.0173)	.134 (.0167)
		GPR-random 1	.136 (.0178)	.134 (.0172)	.135 (.0173)	.134 (.0167)
		GPR-random 10	.136 (.0178)	.134 (.0172)	.135 (.0173)	.134 (.0167)
	300	SPSS	.070 (.0029)	.070 (.0028)	.070 (.0029)	.070 (.0028)
		GPR-unrotated	.070 (.0037)	.070 (.0028)	.070 (.0037)	.070 (.0028)
		GPR-random 1	.070 (.0029)	.070 (.0028)	.070 (.0029)	.070 (.0028)
		GPR-random 10	.070 (.0029)	.070 (.0028)	.070 (.0029)	.070 (.0028)
12	100	SPSS	.138 (.0143)	.137 (.0140)	.138 (.0142)	.137 (.0140)
		GPR-unrotated	.139 (.0149)	.138 (.0146)	.138 (.0143)	.137 (.0145)
		GPR-random 1	.139 (.0149)	.138 (.0146)	.138 (.0143)	.137 (.0145)
		GPR-random 10	.139 (.0149)	.138 (.0146)	.138 (.0143)	.137 (.0145)
	300	SPSS	.071 (.0023)	.071 (.0023)	.071 (.0023)	.071 (.0023)
		GPR-unrotated	.071 (.0023)	.071 (.0023)	.071 (.0023)	.071 (.0023)
		GPR-random 1	.071 (.0023)	.071 (.0023)	.071 (.0023)	.071 (.0023)
		GPR-random 10	.071 (.0023)	.071 (.0023)	.071 (.0023)	.071 (.0023)

Note. GPR = gradient-projection based rotation. *k* = number of components. Rotation after principal component analysis of simulated data.

Supplementary Table 8

Varimax criterion for component rotation by SPSS-Varimax and GPR-Varimax for the single-optimum model and 8 variables per component – Means and standard deviations of 1,000 samples

			25 iterations		250 iterations	
k	N	Rotation method	No Kaiser	With Kaiser	No Kaiser	With Kaiser
3	100	SPSS	.0267 (.00314)	.0266 (.00315)	.0267 (.00314)	.0226 (.00315)
		GPR-unrotated	.0267 (.00326)	.0268 (.00349)	.0267 (.00314)	.0226 (.00315)
		GPR-random 1	.0267 (.00314)	.0266 (.00315)	.0267 (.00314)	.0226 (.00315)
		GPR-random 10	.0267 (.00314)	.0266 (.00315)	.0267 (.00314)	.0226 (.00315)
	300	SPSS	.0263 (.00176)	.0263 (.00176)	.0263 (.00176)	.0263 (.00176)
		GPR-unrotated	.0263 (.00176)	.0263 (.00176)	.0263 (.00176)	.0263 (.00176)
		GPR-random 1	.0263 (.00176)	.0263 (.00176)	.0263 (.00176)	.0263 (.00176)
		GPR-random 10	.0263 (.00176)	.0263 (.00176)	.0263 (.00176)	.0263 (.00176)
6	100	SPSS	.0153 (.00140)	.0152 (.00141)	.0153 (.00140)	.0152 (.00141)
		GPR-unrotated	.0153 (.00140)	.0152 (.00141)	.0153 (.00140)	.0152 (.00141)
		GPR-random 1	.0153 (.00140)	.0152 (.00141)	.0153 (.00140)	.0152 (.00141)
		GPR-random 10	.0153 (.00140)	.0152 (.00141)	.0153 (.00140)	.0152 (.00141)
	300	SPSS	.0160 (.00080)	.0159 (.00080)	.0160 (.00080)	.0159 (.00080)
		GPR-unrotated	.0160 (.00080)	.0159 (.00080)	.0160 (.00080)	.0159 (.00080)
		GPR-random 1	.0160 (.00080)	.0159 (.00080)	.0160 (.00080)	.0159 (.00080)
		GPR-random 10	.0160 (.00080)	.0159 (.00080)	.0160 (.00080)	.0159 (.00080)
9	100	SPSS	.0100 (.00088)	.0099 (.00089)	.0100 (.00088)	.0099 (.00089)
		GPR-unrotated	.0100 (.00088)	.0100 (.00088)	.0100 (.00088)	.0100 (.00089)
		GPR-random 1	.0100 (.00089)	.0100 (.00088)	.0100 (.00089)	.0100 (.00089)
		GPR-random 10	.0100 (.00089)	.0100 (.00088)	.0100 (.00089)	.0100 (.00089)
	300	SPSS	.0111 (.00048)	.0110 (.00048)	.0111 (.00048)	.0110 (.00048)
		GPR-unrotated	.0111 (.00048)	.0110 (.00048)	.0111 (.00048)	.0110 (.00048)
		GPR-random 1	.0111 (.00048)	.0110 (.00048)	.0111 (.00048)	.0110 (.00048)
		GPR-random 10	.0111 (.00048)	.0110 (.00048)	.0111 (.00048)	.0110 (.00048)
12	100	SPSS	.0072 (.00055)	.0072 (.00055)	.0072 (.00055)	.0072 (.00056)
		GPR-unrotated	.0072 (.00054)	.0072 (.00055)	.0072 (.00054)	.0072 (.00056)
		GPR-random 1	.0072 (.00056)	.0072 (.00055)	.0072 (.00056)	.0072 (.00056)
		GPR-random 10	.0072 (.00056)	.0072 (.00055)	.0072 (.00056)	.0072 (.00056)
	300	SPSS	.0083 (.00032)	.0083 (.00032)	.0083 (.00032)	.0083 (.00032)
		GPR-unrotated	.0083 (.00032)	.0083 (.00032)	.0083 (.00032)	.0083 (.00032)
		GPR-random 1	.0083 (.00032)	.0083 (.00032)	.0083 (.00032)	.0083 (.00032)
		GPR-random 10	.0083 (.00032)	.0083 (.00032)	.0083 (.00032)	.0083 (.00032)

Note. GPR = gradient-projection based rotation. *k* = number of components. Rotation after principal component analysis of simulated data.

Supplementary Table 9

Mean congruence with population components for component rotation by SPSS-Varimax and GPR-Varimax for the single-optimum model and 8 variables per component – Means and standard deviations of 1,000 samples

			25 iterations		250 iterations	
k	N	Rotation method	No Kaiser	With Kaiser	No Kaiser	With Kaiser
3	100	SPSS	.951 (.0131)	.951 (.0130)	.951 (.0131)	.951 (.0130)
		GPR-unrotated	.949 (.0162)	.950 (.0167)	.951 (.0131)	.951 (.0130)
		GPR-random 1	.951 (.0131)	.951 (.0130)	.951 (.0131)	.951 (.0130)
		GPR-random 10	.951 (.0131)	.951 (.0130)	.951 (.0131)	.951 (.0130)
	300	SPSS	.984 (.0031)	.984 (.0031)	.984 (.0031)	.984 (.0031)
		GPR-unrotated	.984 (.0031)	.984 (.0031)	.984 (.0031)	.984 (.0031)
		GPR-random 1	.984 (.0031)	.984 (.0031)	.984 (.0031)	.984 (.0031)
		GPR-random 10	.984 (.0031)	.984 (.0031)	.984 (.0031)	.984 (.0031)
6	100	SPSS	.893 (.0174)	.894 (.0172)	.893 (.0174)	.894 (.0172)
		GPR-unrotated	.893 (.0174)	.894 (.0172)	.893 (.0174)	.894 (.0172)
		GPR-random 1	.893 (.0174)	.894 (.0172)	.893 (.0174)	.894 (.0172)
		GPR-random 10	.893 (.0174)	.894 (.0172)	.893 (.0174)	.894 (.0172)
	300	SPSS	.965 (.0036)	.965 (.0036)	.965 (.0036)	.965 (.0036)
		GPR-unrotated	.965 (.0036)	.965 (.0036)	.965 (.0036)	.965 (.0036)
		GPR-random 1	.965 (.0036)	.965 (.0036)	.965 (.0036)	.965 (.0036)
		GPR-random 10	.965 (.0036)	.965 (.0036)	.965 (.0036)	.965 (.0036)
9	100	SPSS	.832 (.0270)	.833 (.0268)	.832 (.0270)	.833 (.0267)
		GPR-unrotated	.832 (.0271)	.833 (.0269)	.832 (.0270)	.833 (.0268)
		GPR-random 1	.832 (.0271)	.833 (.0269)	.832 (.0270)	.833 (.0268)
		GPR-random 10	.832 (.0270)	.833 (.0269)	.832 (.0270)	.833 (.0268)
	300	SPSS	.946 (.0039)	.947 (.0039)	.946 (.0039)	.947 (.0039)
		GPR-unrotated	.946 (.0039)	.947 (.0039)	.946 (.0039)	.947 (.0039)
		GPR-random 1	.946 (.0039)	.947 (.0039)	.946 (.0039)	.947 (.0039)
		GPR-random 10	.946 (.0039)	.947 (.0039)	.946 (.0039)	.947 (.0039)
12	100	SPSS	.775 (.0269)	.777 (.0268)	.775 (.0269)	.777 (.0268)
		GPR-unrotated	.774 (.0275)	.777 (.0271)	.775 (.0270)	.777 (.0269)
		GPR-random 1	.774 (.0271)	.777 (.0271)	.775 (.0269)	.777 (.0269)
		GPR-random 10	.775 (.0267)	.777 (.0271)	.775 (.0267)	.777 (.0269)
	300	SPSS	.928 (.0043)	.928 (.0043)	.928 (.0043)	.928 (.0043)
		GPR-unrotated	.928 (.0046)	.928 (.0043)	.928 (.0046)	.928 (.0043)
		GPR-random 1	.928 (.0043)	.928 (.0043)	.928 (.0043)	.928 (.0043)
		GPR-random 10	.928 (.0043)	.928 (.0043)	.928 (.0043)	.928 (.0043)

Note. GPR = gradient-projection based rotation. *k* = number of components. Rotation after principal component analysis of simulated data.

Supplementary Table 10

RMSE as deviation from population components for rotation by SPSS-Varimax and GPR-Varimax for the single-optimum model and 8 variables per component – Means and standard deviations of 1,000 samples

			25 iterations		250 iterations	
k	N	Rotation method	No Kaiser	With Kaiser	No Kaiser	With Kaiser
3	100	SPSS	.108 (.0127)	.108 (.0126)	.108 (.0127)	.108 (.0126)
		GPR-unrotated	.110 (.0150)	.109 (.0155)	.108 (.0127)	.108 (.0126)
		GPR-random 1	.108 (.0127)	.108 (.0126)	.108 (.0127)	.108 (.0126)
		GPR-random 10	.108 (.0127)	.108 (.0126)	.108 (.0127)	.108 (.0126)
	300	SPSS	.061 (.0057)	.061 (.0057)	.061 (.0057)	.061 (.0057)
		GPR-unrotated	.061 (.0057)	.061 (.0057)	.061 (.0057)	.061 (.0057)
		GPR-random 1	.061 (.0057)	.061 (.0057)	.061 (.0057)	.061 (.0057)
		GPR-random 10	.061 (.0057)	.061 (.0057)	.061 (.0057)	.061 (.0057)
6	100	SPSS	.116 (.0093)	.115 (.0093)	.116 (.0093)	.115 (.0093)
		GPR-unrotated	.116 (.0093)	.115 (.0093)	.116 (.0093)	.115 (.0093)
		GPR-random 1	.116 (.0093)	.115 (.0093)	.116 (.0093)	.115 (.0093)
		GPR-random 10	.116 (.0093)	.115 (.0093)	.116 (.0093)	.115 (.0093)
	300	SPSS	.065 (.0031)	.064 (.0030)	.065 (.0031)	.064 (.0030)
		GPR-unrotated	.065 (.0031)	.064 (.0030)	.065 (.0031)	.064 (.0030)
		GPR-random 1	.065 (.0031)	.064 (.0030)	.065 (.0031)	.064 (.0030)
		GPR-random 10	.065 (.0031)	.064 (.0030)	.065 (.0031)	.064 (.0030)
9	100	SPSS	.122 (.0116)	.121 (.0107)	.122 (.0115)	.121 (.0107)
		GPR-unrotated	.122 (.0120)	.121 (.0111)	.122 (.0116)	.121 (.0107)
		GPR-random 1	.122 (.0117)	.121 (.0111)	.122 (.0115)	.121 (.0107)
		GPR-random 10	.122 (.0114)	.121 (.0111)	.122 (.0115)	.121 (.0107)
	300	SPSS	.066 (.0021)	.066 (.0021)	.066 (.0021)	.066 (.0021)
		GPR-unrotated	.066 (.0021)	.066 (.0021)	.066 (.0021)	.066 (.0021)
		GPR-random 1	.066 (.0021)	.066 (.0021)	.066 (.0021)	.066 (.0021)
		GPR-random 10	.066 (.0021)	.066 (.0021)	.066 (.0021)	.066 (.0021)
12	100	SPSS	.126 (.0116)	.125 (.0113)	.126 (.0117)	.125 (.0112)
		GPR-unrotated	.127 (.0120)	.126 (.0113)	.127 (.0119)	.125 (.0114)
		GPR-random 1	.127 (.0120)	.126 (.0113)	.126 (.0116)	.125 (.0114)
		GPR-random 10	.126 (.0115)	.126 (.0113)	.126 (.0115)	.125 (.0114)
	300	SPSS	.066 (.0018)	.066 (.0018)	.066 (.0018)	.066 (.0018)
		GPR-unrotated	.066 (.0024)	.066 (.0018)	.066 (.0024)	.066 (.0018)
		GPR-random 1	.066 (.0018)	.066 (.0018)	.066 (.0018)	.066 (.0018)
		GPR-random 10	.066 (.0018)	.066 (.0018)	.066 (.0018)	.066 (.0018)

Note. GPR = gradient-projection based rotation. *k* = number of components. Rotation after principal component analysis of simulated data.

Supplementary Table 11

Varimax criterion for component rotation by SPSS-Varimax and GPR-Varimax for the double-optimum model – Means and standard deviations of 1,000 samples

			25 iterations		250 iterations	
k	N	Rotation method	No Kaiser	With Kaiser	No Kaiser	With Kaiser
3	100	SPSS	.0227 (.00259)	.0224 (.00262)	.0227 (.00259)	.0224 (.00262)
		GPR-unrotated	.0227 (.00259)	.0224 (.00261)	.0227 (.00259)	.0224 (.00261)
		GPR-random 1	.0227 (.00259)	.0224 (.00262)	.0227 (.00259)	.0224 (.00262)
		GPR-random 10	.0227 (.00259)	.0224 (.00261)	.0227 (.00259)	.0224 (.00262)
	300	SPSS	.0212 (.00143)	.0209 (.00143)	.0212 (.00143)	.0209 (.00143)
		GPR-unrotated	.0212 (.00144)	.0209 (.00144)	.0212 (.00143)	.0209 (.00144)
		GPR-random 1	.0212 (.00144)	.0209 (.00144)	.0212 (.00143)	.0209 (.00143)
		GPR-random 10	.0212 (.00143)	.0210 (.00142)	.0212 (.00143)	.0209 (.00142)
6	100	SPSS	.0136 (.00117)	.0135 (.00119)	.0136 (.00117)	.0135 (.00119)
		GPR-unrotated	.0136 (.00117)	.0135 (.00119)	.0136 (.00117)	.0135 (.00119)
		GPR-random 1	.0136 (.00116)	.0135 (.00119)	.0136 (.00116)	.0135 (.00119)
		GPR-random 10	.0136 (.00116)	.0135 (.00119)	.0136 (.00116)	.0135 (.00119)
	300	SPSS	.0135 (.00069)	.0133 (.00068)	.0135 (.00069)	.0133 (.00069)
		GPR-unrotated	.0135 (.00069)	.0133 (.00068)	.0135 (.00069)	.0133 (.00069)
		GPR-random 1	.0135 (.00069)	.0133 (.00069)	.0135 (.00069)	.0133 (.00068)
		GPR-random 10	.0135 (.00069)	.0134 (.00068)	.0135 (.00069)	.0134 (.00068)
9	100	SPSS	.0091 (.00075)	.0090 (.00076)	.0091 (.00075)	.0090 (.00076)
		GPR-unrotated	.0091 (.00075)	.0090 (.00075)	.0091 (.00075)	.0090 (.00075)
		GPR-random 1	.0091 (.00075)	.0090 (.00075)	.0091 (.00075)	.0090 (.00075)
		GPR-random 10	.0091 (.00075)	.0090 (.00075)	.0091 (.00075)	.0090 (.00075)
	300	SPSS	.0094 (.00041)	.0094 (.00042)	.0094 (.00041)	.0094 (.00042)
		GPR-unrotated	.0094 (.00041)	.0094 (.00042)	.0094 (.00041)	.0094 (.00042)
		GPR-random 1	.0094 (.00041)	.0094 (.00041)	.0094 (.00041)	.0094 (.00041)
		GPR-random 10	.0094 (.00041)	.0094 (.00041)	.0094 (.00041)	.0094 (.00041)
12	100	SPSS	.0067 (.00049)	.0066 (.00051)	.0067 (.00049)	.0066 (.00050)
		GPR-unrotated	.0067 (.00049)	.0066 (.00051)	.0067 (.00049)	.0066 (.00050)
		GPR-random 1	.0067 (.00049)	.0066 (.00051)	.0067 (.00049)	.0066 (.00050)
		GPR-random 10	.0067 (.00049)	.0066 (.00051)	.0067 (.00049)	.0066 (.00050)
	300	SPSS	.0072 (.00027)	.0071 (.00028)	.0072 (.00027)	.0071 (.00028)
		GPR-unrotated	.0072 (.00027)	.0071 (.00028)	.0072 (.00027)	.0071 (.00028)
		GPR-random 1	.0072 (.00027)	.0071 (.00028)	.0072 (.00027)	.0071 (.00028)
		GPR-random 10	.0072 (.00027)	.0071 (.00027)	.0072 (.00027)	.0071 (.00028)

Note. GPR = gradient-projection based rotation. *k* = number of components. Rotation after principal component analysis of simulated data.

Supplementary Table 12

Mean congruence with population components for component rotation by SPSS-Varimax and GPR-Varimax for the double-optimum model – Means and standard deviations of 1,000 samples

			25 iterations		250 iterations	
k	N	Rotation method	No Kaiser	With Kaiser	No Kaiser	With Kaiser
3	100	SPSS	.910 (.0470)	.883 (.0659)	.910 (.0470)	.883 (.0659)
		GPR-unrotated	.910 (.0474)	.884 (.0656)	.910 (.0470)	.884 (.0659)
		GPR-random 1	.910 (.0469)	.900 (.0463)	.910 (.0470)	.900 (.0462)
		GPR-random 10	.910 (.0470)	.900 (.0466)	.910 (.0470)	.900 (.0462)
	300	SPSS	.957 (.0370)	.906 (.0755)	.957 (.0370)	.906 (.0755)
		GPR-unrotated	.958 (.0366)	.908 (.0749)	.957 (.0369)	.906 (.0755)
		GPR-random 1	.958 (.0363)	.933 (.0366)	.957 (.0368)	.935 (.0352)
		GPR-random 10	.957 (.0369)	.942 (.0345)	.957 (.0371)	.936 (.0344)
6	100	SPSS	.853 (.0339)	.830 (.0444)	.853 (.0339)	.830 (.0444)
		GPR-unrotated	.853 (.0340)	.830 (.0442)	.853 (.0339)	.830 (.0444)
		GPR-random 1	.854 (.0353)	.830 (.0442)	.853 (.0338)	.830 (.0444)
		GPR-random 10	.853 (.0339)	.830 (.0442)	.853 (.0339)	.830 (.0444)
	300	SPSS	.940 (.0234)	.904 (.0405)	.940 (.0234)	.890 (.0491)
		GPR-unrotated	.940 (.0246)	.904 (.0406)	.940 (.0236)	.890 (.0489)
		GPR-random 1	.940 (.0242)	.920 (.0264)	.940 (.0236)	.920 (.0263)
		GPR-random 10	.940 (.0235)	.927 (.0234)	.940 (.0234)	.922 (.0256)
9	100	SPSS	.794 (.0354)	.777 (.0364)	.794 (.0355)	.777 (.0364)
		GPR-unrotated	.793 (.0355)	.776 (.0366)	.794 (.0354)	.777 (.0366)
		GPR-random 1	.793 (.0355)	.776 (.0366)	.794 (.0354)	.777 (.0366)
		GPR-random 10	.793 (.0355)	.776 (.0366)	.794 (.0354)	.777 (.0366)
	300	SPSS	.922 (.0191)	.876 (.0386)	.922 (.0191)	.878 (.0402)
		GPR-unrotated	.921 (.0193)	.876 (.0385)	.921 (.0192)	.878 (.0403)
		GPR-random 1	.921 (.0195)	.903 (.0212)	.921 (.0192)	.904 (.0202)
		GPR-random 10	.922 (.0190)	.910 (.0193)	.922 (.0190)	.906 (.0202)
12	100	SPSS	.738 (.0346)	.727 (.0337)	.737 (.0347)	.725 (.0341)
		GPR-unrotated	.737 (.0345)	.726 (.0332)	.738 (.0346)	.725 (.0341)
		GPR-random 1	.737 (.0345)	.726 (.0332)	.738 (.0346)	.725 (.0341)
		GPR-random 10	.737 (.0345)	.726 (.0332)	.738 (.0346)	.725 (.0341)
	300	SPSS	.902 (.0181)	.861 (.0328)	.902 (.0181)	.860 (.0331)
		GPR-unrotated	.902 (.0183)	.861 (.0323)	.902 (.0183)	.860 (.0331)
		GPR-random 1	.902 (.0184)	.887 (.0191)	.902 (.0184)	.887 (.0186)
		GPR-random 10	.902 (.0183)	.892 (.0179)	.902 (.0182)	.889 (.0180)

Note. GPR = gradient-projection based rotation. *k* = number of components. Rotation after principal component analysis of simulated data.

Supplementary Table 13

RMSE as deviation from population components for rotation by SPSS-Varimax and GPR-Varimax for the double-optimum model – Means and standard deviations of 1,000 samples

			25 iterations		250 iterations	
k	N	Rotation method	No Kaiser	With Kaiser	No Kaiser	With Kaiser
3	100	SPSS	.378 (.1454)	.379 (.1437)	.378 (.1476)	.379 (.1436)
		GPR-unrotated	.377 (.1461)	.378 (.1435)	.379 (.1475)	.378 (.1439)
		GPR-random 1	.377 (.1460)	.386 (.1380)	.378 (.1476)	.379 (.1454)
		GPR-random 10	.377 (.1455)	.385 (.1388)	.378 (.1476)	.379 (.1454)
	300	SPSS	.360 (.1648)	.359 (.1519)	.360 (.1648)	.359 (.1519)
		GPR-unrotated	.359 (.1657)	.359 (.1519)	.359 (.1653)	.361 (.1515)
		GPR-random 1	.361 (.1658)	.359 (.1519)	.360 (.1652)	.368 (.1505)
		GPR-random 10	.359 (.1650)	.359 (.1519)	.360 (.1649)	.368 (.1514)
6	100	SPSS	.288 (.0608)	.287 (.0621)	.288 (.0608)	.287 (.0621)
		GPR-unrotated	.288 (.0609)	.287 (.0621)	.288 (.0609)	.286 (.0626)
		GPR-random 1	.288 (.0611)	.287 (.0621)	.288 (.0611)	.286 (.0626)
		GPR-random 10	.288 (.0606)	.287 (.0621)	.288 (.0607)	.286 (.0626)
	300	SPSS	.272 (.0726)	.271 (.0704)	.271 (.0741)	.277 (.0636)
		GPR-unrotated	.272 (.0731)	.273 (.0694)	.271 (.0737)	.277 (.0632)
		GPR-random 1	.272 (.0727)	.273 (.0698)	.271 (.0735)	.273 (.0700)
		GPR-random 10	.272 (.0731)	.273 (.0702)	.271 (.0736)	.274 (.0700)
9	100	SPSS	.246 (.0375)	.247 (.0358)	.246 (.0375)	.247 (.0359)
		GPR-unrotated	.246 (.0378)	.247 (.0359)	.247 (.0376)	.247 (.0360)
		GPR-random 1	.246 (.0378)	.247 (.0359)	.247 (.0376)	.247 (.0360)
		GPR-random 10	.246 (.0378)	.247 (.0359)	.247 (.0376)	.247 (.0360)
	300	SPSS	.229 (.0440)	.230 (.0420)	.229 (.0437)	.229 (.0424)
		GPR-unrotated	.229 (.0443)	.231 (.0413)	.229 (.0436)	.229 (.0422)
		GPR-random 1	.229 (.0439)	.229 (.0439)	.229 (.0436)	.228 (.0432)
		GPR-random 10	.229 (.0438)	.229 (.0433)	.229 (.0434)	.229 (.0433)
12	100	SPSS	.222 (.0254)	.220 (.0256)	.220 (.0269)	.221 (.0251)
		GPR-unrotated	.223 (.0254)	.220 (.0255)	.220 (.0270)	.221 (.0253)
		GPR-random 1	.223 (.0254)	.220 (.0255)	.220 (.0270)	.221 (.0253)
		GPR-random 10	.223 (.0254)	.220 (.0255)	.220 (.0270)	.221 (.0253)
	300	SPSS	.201 (.0338)	.202 (.0302)	.202 (.0324)	.201 (.0308)
		GPR-unrotated	.202 (.0332)	.202 (.0303)	.202 (.0323)	.201 (.0307)
		GPR-random 1	.201 (.0337)	.201 (.0312)	.202 (.0322)	.201 (.0314)
		GPR-random 10	.202 (.0336)	.201 (.0317)	.202 (.0323)	.201 (.0314)

Note. GPR = gradient-projection based rotation. *k* = number of components. Rotation after principal component analysis of simulated data.

Supplementary Table 14

Empirical example: Inter-correlations of 17 knowledge items from three knowledge domains (n = 397)

	G/h 1	G/h 2	G/h 3	G/h 4	G/h 5	G/h 6	Sc 1	Sc 2	Sc 3	Sc 4	Sc 5	C 1	C 2	C 3	C 4	C 5	C 6
G/h 1	1.000																
G/h 2	.057	1.000															
G/h 3	.097	.178	1.000														
G/h 4	.154	.130	.182	1.000													
G/h 5	.079	.089	.217	.067	1.000												
G/h 6	.092	.114	.154	.067	.101	1.000											
Sc 1	.057	.024	.074	-.009	.051	.087	1.000										
Sc 2	.108	.091	.193	.030	.108	.091	.162	1.000									
Sc 3	.097	.055	.101	.040	.159	.155	.184	.193	1.000								
Sc 4	.017	-.007	-.031	-.006	.014	.045	.181	.216	.322	1.000							
Sc 5	Sc 1	-.019	.019	.040	.089	.039	.127	.062	.229	.322	1.000						
C 1	.061	.052	.039	-.005	.104	.121	.081	.069	.084	.009	.043	1.000					
C 2	-.034	.009	.133	-.042	.057	.126	-.012	.177	.054	.096	.094	.061	1.000				
C 3	-.001	.046	.024	.013	-.014	.156	.089	.074	.089	.143	.049	.096	.044	1.000			
C 4	.058	.014	.115	.032	.109	.152	.131	.012	.150	.048	-.015	.118	.139	.070	1.000		
C 5	.064	.013	.136	.084	.149	.108	.057	.108	.139	.059	.081	.177	.197	.059	.122	1.000	
C 6	-.024	.066	.026	-.008	.003	.138	.053	.158	.124	.092	.049	-.065	.138	.097	.115	.032	1.000

Note. G/h = Geography/history. Sc = Science. C = Culture.
